# C-terminal binding protein-2 triggers CYR61-induced metastatic dissemination of osteosarcoma in a non-hypoxic microenvironment

**DOI:** 10.1186/s13046-025-03350-6

**Published:** 2025-03-05

**Authors:** Laura Di Patria, Nadia Habel, Robert Olaso, Romain Fernandes, Catherine Brenner, Bojana Stefanovska, Olivia Fromigue

**Affiliations:** 1 Inserm UMR981, Gustave Roussy Cancer Campus, Molecular Predictors and New Targets in Oncology, Université Paris Saclay, 39 Rue Camille Desmoulins, Villejuif, F-94805 France; 2 Department of Biomolecular Sciences, University of Urbino Carlo Bo, Urbino, Italy; 3 Université Paris Saclay, CEA, Centre National de Recherche en Génomique Humaine (CNRGH), Evry, France; 4 CNRS UMR9018, Gustave Roussy, Metabolic and Systemic Aspects of Oncogenesis for New Therapeutic Approaches, Université Paris Saclay, Villejuif, France; 5 Present Address : Centre de Traitement de L’Information Génétique (CTIG), INRAE, Jouy en Josas, France; 6 Present Address: Department of Biochemistry and Structural Biology, Howard Hughes Medical Institute, University of Texas Health San Antonio, San Antonio, TX USA

**Keywords:** Cysteine-rich angiogenic protein 61, C-terminal Binding Protein, Hypoxia, Metastasis, Invasion, Bone tumor

## Abstract

**Background:**

Osteosarcoma is the most prevalent cancer-related bone disease diagnosed in the pediatric age group. The rapid development of metastatic lesions and resistance to chemotherapy remain major mechanisms responsible for the failure of treatments and poor outcome. We established that the expression level of Cysteine-rich protein 61 (CYR61/CCN1) correlates to tumor neo-vascularization and dissemination in preclinical and clinical osteosarcoma samples. The aim of this study was to investigate the CYR61-related mechanisms leading to the acquisition of metastatic capacity by osteosarcoma cells.

**Methods:**

Transcriptomic data issued from RNA-seq were subjected to pathways and gene set enrichment analyses. Murine and human cell lines with overexpressed or downregulated C-terminal Binding protein 2 (CtBP2) were established by lentiviral transduction. Cell metabolic activity was assessed by Seahorse XF Analyzer; cell replication rate by BrdU incorporation assay; stemness by clonogenicity assay and RT-qPCR detection of markers; cell migration by wound healing assay and Boyden chambers system; cell invasion using Matrigel coated Boyden chambers or fluorescence microscopy of Matrigel embedded 3D spheroids. FFPE samples derived from syngeneic tumor cells grafts into BALB/c mice were analyzed by IHC. The protein interactome was predicted in silico using the STRING database.

**Results:**

GSEA revealed that CYR61 modulate the transcription process. The in vitro expression level of *CtBP2* and *Cyr61* correlated positively in a panel of osteosarcoma cell lines. In silico analysis of protein–protein interaction network revealed a link with stemness markers. Variations in CtBP2 expression levels influenced stemness markers expression levels, cell clonogenicity, cell migration, Matrix Metalloproteinase activity and cell invasion. Surprisingly, while induction of CtBP2 expression under CYR61 correlated with the metastatic dissemination process in vivo, it occurred only at the invasive front of tumors. Hypoxic conditions in central tumor region interfered with CtBP2 induction of expression.

**Conclusions:**

Our findings identify for the first time that CtBP2 acts as a required critical inducing factor in the CYR61-related metastatic progression of osteosarcoma, by favoring cell migration and invasiveness. Moreover, we demonstrate that while CtBP2 is a downstream transcriptional target of CYR61 signaling cascade, it occurs only under non-hypoxic conditions. The present study suggests that CtBP2 may represent a potential pivotal target for therapeutic management of metastases spreading in osteosarcoma.

**Supplementary Information:**

The online version contains supplementary material available at 10.1186/s13046-025-03350-6.

## Background

Osteosarcoma is the most common malignant skeletal tumor and most often affects children and young adults. These bone tumors frequently spread locally and develop metastases into distant organs (predominately to the lungs) [1]. The therapeutic protocol consists of neo-adjuvant chemotherapy, surgical removal of the tumor if the location is accessible, then multi-agent chemotherapy regimens. The 5-year event free survival (EFS) and overall survival (OS) rates for patients with localized osteosarcoma are 64% and 90%, respectively [2]. In contrast, patients with detectable metastases at diagnosis or with recurrent disease have worse overall prognosis with 5-year EFS and OS rates of only 25% and 21%, respectively. The improvement of clinical outcome for patients with poor prognosis is challenging, and requires the establishment of new therapeutic strategies to prevent the development of metastatic disease.

Several key processes are required for the successful establishment of distant metastasis. Thereby, early in the metastatic cascade, cancer cells must acquire invasive properties, and gain access to the blood or lymphatic vascular systems. We showed previously that statins induce anti-tumor effects on osteosarcoma cells [3–5], and lead to the down-regulation of cysteine rich protein 61 (CYR61/CCN1) expression level in these tumor cells [6]. The immediate-early gene *CYR61* encodes a member of the extracellular matrix-associated CCN family of six homologous cysteine-rich proteins in vertebrates comprising connective tissue growth factor (CTGF), nephroblastoma overexpressed (NOV), and Wnt-induced secreted proteins (WISPs). This family of proteins, with CYR61 as forerunner, are involved in multiple physiological functions such as skeletal and cardiovascular development and injury repair [7–10]. In different solid cancer types, CYR61 promotes tumor growth and vascularization as well as cell invasiveness and metastasis [11–15]. We established that CYR61 expression levels correlate with the metastatic capacities of osteosarcoma tumor cells both in vitro and in preclinical murine models, and correlate positively with tumor grade in patients [6]. Our previous data support the main role of CYR61 in tumor vascularization through the promotion of neo-angiogenesis [6, 16] and remodeling/destruction of the extracellular matrix surrounding the primary tumor [6, 16]. In addition, we demonstrated that *CYR61* silencing significantly reduces in vitro cell migration potential and expression of pro-angiogenic factors, but also reduces in vivo tumor neovascularization and lung metastases occurrence [6, 16, 17]. The overexpression of CYR61 in cancer cells is commonly associated with epithelial-to-mesenchymal transition (EMT) [18–22]. In fact, when progressing to a more malignant state, tumor cells frequently lose some of their epithelial characteristics and acquire properties of migrating cells [23]. We have reported a process similar to EMT in osteosarcoma cells despite their mesenchymal origin, and demonstrated that *CYR61* silencing reinforces the mesenchymal phenotype [17]. Globally, our data established CYR61 as promising key marker of tumor dissemination thus representing an appealing therapeutic target in metastatic osteosarcoma. However, its ubiquitous nature and its multiple physiological roles are the main obstacles for the development of targeted therapeutic strategies. A better characterization of the pro-metastatic cascade reliant on CYR61 is crucial to refine the therapeutic options.

In the present study, we confirmed the effect of CYR61 on dissemination abilities of osteosarcoma cells, and identified a new essential downstream effector. Our results indicate that the expression of the transcriptional co-repressor CtBP2 (Carboxyl-terminal Binding Protein 2) is induced by CYR61 at a transcriptional level. Furthermore, we highlighted a spatial regulation of CtBP2 induction, mainly dependent on oxygen levels. Our results demonstrate that CtBP2 is an essential component of the CYR61-dependent pro-metastatic cascade in osteosarcoma cells.

## Materials and methods

### Maintenance of osteosarcoma cell lines

The murine K7M2 cell line, and the human 143B, HOS, MG-63, SaOS-2, and U-2 OS cell lines were originally obtained from ATCC (LGC Standards sarl, Molsheim, France). The human IOR/OS18 cell line was kindly provided by Pr M. Serra (Istituti Ortopedici Rizzoli, Bologna; Italy) [24]. The human CAL72 cell line was kindly provided by Dr N. Rochet (Institute of Biology Valrose, Nice, France) [25]. The human OHS-4 cell line was kindly provided by Drs Fournier and Price (University of California, San Diego, CA, USA) [26].The human embryonic kidney cells HEK293T were obtained from Pr S Postel-Vinay (UMR981, Gustave Roussy, France).

All cell lines were routinely grown in GlutaMAX™-containing high glucose Dulbecco's Modified Eagles Medium (DMEM; Thermo Fisher Scientific, Courtabœuf, France) supplemented with 10% heat inactivated fetal bovine serum (FBS), at 37 °C in a saturated humidity atmosphere (5% CO_2_ and 95% air). Culture media were changed three times a week, and regularly tested for mycoplasma contamination (PCR-based assay from Minerva-Biolabs, Berlin, Germany).

### Generation of stable cell lines by lentiviral vector transduction

The K7M2 cell lines stably modified for CYR61 were established as previously described [6].

Ready-to-use lentiviral particles containing a vector encoding human or murine CtBP2 shRNA sequences or non-targeting (control) shRNA sequences were purchased from Santa Cruz Biotechnology (Santa Cruz, CA, USA). Briefly, sub-confluent target cells were incubated with particles and 4 µg/mL polybrene (Hexadimethrine bromide; Sigma-Aldrich, Lyon, France) for 18 h, washed once with medium, and cultured for 48 h. Non-transduced cells were eliminated by puromycin dihydrochloride selection (10 µg/mL; Sigma-Aldrich) for 3 days. Viable cells were routinely maintained in complete medium.

Lentiviral vectors for CtBP2 over-expression in mammalian cells were generated by Gateway cloning. The donor vector pDONR223-CtBP2 was purchased from the central repository DNASU (Arizona State University, Tempe, AZ, USA), and the destination lentiviral empty vector pLX303 was purchased from Addgene (Cambridge, MA, USA). The sub-cloning of the CtBP2 sequence into pLX303 lentiviral vector was performed by site-specific recombination using LR clonase-II (Thermo Fisher Scientific), according to the manufacturer’s recommendations. Particles were produced using human embryonic kidney cells HEK293T as previously described [27]. Supernatants were collected after 48 h incubation, centrifuged at 200 g to remove cell debris, and filtered through a 0.45 µm low protein-binding filter (Corning, Bath, UK). Sub-confluent target cells were incubated with the particles-containing supernatant supplemented with 4 µg/mL polybrene for 24 h, washed once with medium, and cultured for 48 h before Blasticidin selection (5 µg/mL; Sigma-Aldrich) for 3 days. Viable cells were routinely maintained in complete medium.

### RNA isolation

Total RNAs were isolated using the RNeasy Mini Kit (Qiagen) according to the manufacturer’s instruction, and stored at −80 °C. RNA quantitation, and quality control were performed on Agilent 2100 Bioanalyzer to select samples exhibiting RNA Integrity Number = 10.

### Library preparation and next-generation sequencing

Total RNA samples (400 ng) were send to Integragen SA (Evry, France) for libraries preparation and sequencing. Libraries are prepared with NEB Next Ultra II Directional RNA Library Prep Kit for Illumina protocol according supplier recommendations. Briefly, the key stages were successively: the purification of PolyA containing mRNA molecules using polyT oligo attached magnetic beads from 100 ng total RNA (with the Magnetic mRNA Isolation Kit from NEB); a fragmentation using divalent cations under elevated temperature to obtain approximately 300 bp pieces; double strand cDNA synthesis and finally Illumina adapters ligation and cDNA library amplification by PCR. Sequencing was carried out on Paired-End 100 bp reads on Illumina NovaSeqX platform. Image analysis and base calling was performed using Illumina Real-Time Analysis with default parameters.

### Data analysis

The raw reads were demultiplexed, QCed and trimmed using FastQC. The high-quality sequences were mapped to the mouse Reference genome assembly (GRCm38/mm10) generating bam format. The number of reads associated to each gene in the GENCODE vM24 reference annotation was obtained using STAR aligner (v2.7.10a). Raw counts for each sample were imported into R environment (v4.0.2). The normalized measures of the proportion of reads that mapped to a gene in each sample (TPM; Transcripts Per Million) were used to compare gene expression between samples.

### Unsupervised analysis

The Bioconductor edgeR package (v3.32.0) was used to import raw counts into R statistical software, and compute normalized log2 CPM (counts per millions of mapped reads) using the TMM (weighted trimmed mean of M-values) as normalization procedure. The normalized expression matrix from the 1000 most variant genes (based on standard deviation) while excluding gender-related genes, Immunoglobulin (Ig) variable chain and T-cell receptor (TcR) genes, was used to classify the samples according to their gene expression patterns using principal component analysis (PCA). PCA was performed by FactoMineR::PCA function with “ncp = 10, scale.unit = FALSE” parameters.

### Differential expression and pathway enrichment analyses

Differential expression analysis was performed using the Bioconductor limma package (v3.46.0) and the voom transformation. To improve the statistical power of the analysis, only genes expressed in at least one sample (TPM > = 0.3) were considered. A normalization factor was calculated based on the geometric mean of the expression levels of housekeeping genes, namely Gapdh, Hprt, Rpl26, Rpl32, and Tbp. Genes that showed at least a fold change |FC|> 1.15 in expression and a q-value (*p*-value corrected by Benjamini–Hochberg procedure) threshold of < = 0.05 were considered for detailed functional analysis. Gene ontology (GO) annotations of differentially expressed genes (DEGs) was performed using the following online databases: Cytoscape v3.10.2 [28], DAVID (Database for Annotation, Visualization and Integrated Discovery) v2021 [29, 30] and Panther classification system v18.0 [31].

### Gene expression correlation in patient samples

The gene expression correlation analyses were performed using the publicly available R2 database (R2: Genomics Analysis and Visualization Platform; http://r2.amc.nl). Three independent cohorts of osteosarcoma samples with gene expression levels were identified, and queried for CYR61 and CtBP2 correlation.

### Protein–protein interaction network

The STRING database (v11.5) was used as Core Data Resource for all known and predicted associations between proteins, including both physical interactions as well as functional associations [32]. Settings included full STRING network, excluding gene fusion from the selection of active interaction sources.

### Reverse Transcription and quantitative Polymerase Chain Reaction (RT-qPCR)

Total RNA (3 µg) were denatured for 10 min at 70 °C then reverse transcribed at 37 °C for 90 min in a buffer containing Superscript II reverse transcriptase (Thermo Fisher Scientific), Dithiothreitol (10 mM), random Hexanucleotides (2.5 µM), and dNTPs mix (0.5 mM). A final step of inactivation of the reverse transcriptase was carried out at 95 °C for 10 min.

Real-time quantitative Polymerase Chain Reaction (qPCR) was carried out on ViiA7 apparatus (Thermo Fisher Scientific) using SYBR Green Master kit (Thermo Fisher Scientific) supplemented with 0.5 µM of specific primers (Supplemental Table 1). After a first step of enzyme activation at 95 °C for 15 min, cDNA were processed for 45 cycles of denaturation (95 °C for 20 s), annealing (58 °C for 20 s) and extension (72 °C for 20 s). Upon run completion, a melting curve analysis was included to assure that only one PCR amplicon was formed. The relative fold gene expressions were estimated using the 2^−∆∆Ct^ method.

### Cell metabolic activity assay

The metabolic activity was evaluated using both colorimetric and luminescent assay. Briefly, MTT reagent (3-(4,5-dimethylthiazol-2-yl)−2,5-diphenyltetrazolium bromide; Sigma Aldrich) was added to the culture medium at a final concentration of 1 mg/mL for the last 1 h of incubation. The absorbance of tetrazolium precipitates solubilized in DMSO was measured at the wavelength of 592 nm. The half-maximal inhibitory concentration (IC_50_ value) was interpolated from sigmoidal dose-responses curves (GraphPad Prism software version 10.4.1 for Windows, Boston, MA, USA; www.graphpad.com).

A quantitation of the ATP present in cell culture, reflecting the proportion of metabolically active cells was determined using the CellTiter-Glo luminescent cell viability assay (Promega). Briefly, cells were seeded at the density of 10,000 cells/cm^2^ in 96-wells plates, and cultured for 48 h. For the assay, the plate was incubated at RT for 15 min before addition of the CellTiter-Glo reagent (1:5 volume ratio), then placed on a rocking platform for 15 min at RT in the dark. Luminescence signal was detected using Spark microplate reader (Tecan, Männedorf, Switzerland).

### Extracellular flux analysis using Seahorse bioscience XFe96 analyzer

The oxygen consumption rate (OCR) and extracellular acidification rate (ECAR) of cells were kinetically determined using the Mito Stress Test kit (Agilent Technologies, Les Ulis, France) at multiple time steps, according to the manufacturer’s instructions. Briefly, the day before the experiment, probes-containing cartridges were hydrated with Seahorse calibrant and incubated in a non-CO_2_ incubator at 37 °C. Then, cells were seeded at a density of 3.10^4 ^cells/cm^2^ in Seahorse 96-well microplates and incubated overnight at 37 °C in a humidified incubator with 5% CO_2_. The four corners of the plate were filed with medium for background correction. The day after, cells were washed with pre-warmed Seahorse medium (phenol red free DMEM medium pH 7.4 supplemented with 10 mM glucose, 1 mM pyruvate and 2 mM glutamine), and incubated at 37 °C in a non-CO_2_ incubator for 45 min prior measurement. OCR was determined at basal level and after automatic injection of oligomycin (1.5 μM), carbonyl cyanide 4-(trifluoromethoxy) phenylhydrazone (FCCP, 1 μM), and rotenone/antimycin A (0.5 μM). For results normalisation, upon completion of the Seahorse XFe96 Flux analysis, cells were stained with Hoechst-33342 (2.5 µg/mL), and cell number per well was determined using the Cell Imaging Multi-Mode Reader Cytation (BioTek).

The glycolytic function was kinetically determined using the Glycolysis Stress Test Kit (Agilent) at multiple time steps, according to the manufacturer’s instructions. Briefly, cells were seeded and processed as for Mito Stress Test except that the assay medium contained no glucose. ECAR was determined at basal level, and after automatic injection of glucose (10 mM), oligomycin (1 µM), and 2-deoxyglucose (2-DG; 50 mM).

### DNA replication assay

The cell proliferation rate was evaluated using the Kit Biotrak ELISA System (GE Healthcare, Orsay, France) according to the manufacturer's instructions. Briefly, BrdU reagent (5-bromo-2'-deoxyuridine) was added to the culture medium at a final concentration of 10 µM for the last 3 h of incubation. The quantification of BrdU incorporation into newly synthesized DNA of the actively proliferating cells was measured at the wavelength of 592 nm.

### Flow cytometry for cell cycle analysis

After harvesting and wash with PBS, cells (2.10^6^) were fixed in ice-cold 70% ethanol while vortexing, and stored at 4 °C overnight. Cells were rinsed twice with PBS, resuspended in PBS containing 1 µg/mL DAPI and 0.1% Triton-X100, and incubated for 30 min in the dark at RT. Cell cycle distribution was measured using CytoFLEX flow cytometer (Becton–Dickinson). Single cell populations were gated according to FSC-A *vs.* SSC-A to select cell-like events then according to FSC-A *vs.* FSC-H to select singlets. The DAPI signal intensity was illustrated as histogram, and the percentage of cells in the various stages of the cell cycle was determined using the cell cycle analysis platform of FlowJo software (v10.9; BD Life Sciences).

### Clonogenic assay

The ability of single cells to grow into colonies was evaluated using an in vitro cell survival assay. Cells were seeded at the density of 100 cells/cm^2^ in 12-wells plates and cultured for 10 days. Cells were then washed with PBS, fixed with 75% ethanol, and stained with crystal violet solution (0.05% in ethanol). The number and size of cell colonies were then evaluated using ImageJ software (v 1.53t; National Institutes of Health, Bethesda, MD, USA).

### Limiting dilution analysis of sphere-forming cells

Cells were seeded in complete medium at limiting dilutions (1,000 or 100 or 10 or 1 cell/well) in 96-well round plates coated with Poly-2-hydroxyethyl methacrylate (12 mg/mL; Sigma-Aldrich) to prevent cell adhesion. After 10 days, wells were examined using an inverted microscope (Eclipse Ti Series, Nikon, Japan), and scored as positive or negative based on the presence or absence of sphere.

### Fluorescence microscopy for mitochondrial topography

Cells were seeded at a density of 5.10^3^ cell/cm^2^ on coverslips disposed in 6-well plates. The day after, cells were gently rinsed twice with pre-warmed serum-free DMEM, incubated at 37 °C for 30 min in serum-free DMEM containing MitoTracker Red CMXRos (200 nM; Thermo Fisher Scientific), washed twice with PBS, and fixed with 1% paraformaldehyde for 10 min at RT. After two gentle washes in PBS, cells were permeabilized with 0.01% Triton X-100 for 3 min at RT, incubated with 3% BSA for 30 min, then with anti-Tom20 antibody (sc17764 at 1:500 dilution in 1.5% BSA; Santa Cruz Biotechnology) for 90 min at RT. After three washes in PBS/1% Tween20, cells were incubated with goat anti-mouse secondary antibody (1/500 in 1.5% BSA; Abberior Star, Göttingen, Germany). Finally, coverslips were rinsed twice with PBS/1% Tween20, and mounted on glass slides using fluoromount medium (Thermo Fisher Scientific). Coverslips were examined under an inverted fluorescence confocal microscope (STED microscope) using a 100 × 1.4 NA oil immersion objective (Ex 640 nm, Em 650–700 nm for Tom20 signal; Ex 561 nm, Em 575–625 nm for MitoTracker signal). The XY pixel size was set to 100 nm, and Z-stacks were acquired with a 400 nm step size.

### Mitochondrial DNA content

Genomic DNA was extracted using the QIAamp DNA Mini Kit (Qiagen), according to the manufacturer’s recommendations. The concentration of DNA was determined using the Qubit fluorometer (Thermo Fisher Scientific). Real-time quantitative PCR was carried out on ViiA7 apparatus (Thermo Fisher Scientific) using SYBR Green Master kit (Thermo Fisher Scientific) supplemented with 0.5 µM of specific primers (Supplemental Table 2) for the mitochondrially encoded NADH dehydrogenase 2 (*mt-Nd2*) gene, and rodent tandem B1 repetitive elements as reference. After a first step of enzyme activation at 95 °C for 15 min, DNA samples were processed for 45 cycles of denaturation (95 °C for 20 s), annealing (60 °C for 45 s) and extension (72 °C for 1 min). Upon run completion, a melting curve analysis was included to assure that only one PCR amplicon was formed. The relative fold gene expressions were estimated using the 2^−∆∆Ct^ method.

### Wound healing assay

The cell motility was evaluated using removable silicone inserts, according to manufacturer’s instructions (Ibidi, Martinsried, Germany). Cell monolayers were cultured for 18 h, fixed in 75% ethanol, and then stained with crystal violet solution (0.05% in ethanol). Recovery of the denuded area was computerized using an inverted digital microscope (EVOS imaging system), and evaluated using ImageJ software package. Lesion area surface at time zero was used as matrix.

### Chemotactic cell migration and invasion assays

The cell migration rate was evaluated using modified Boyden chamber (8 μm pore size Falcon® Transwell inserts, Corning, Boulogne-Billancourt, France). Briefly, 5 × 10^4^ serum-starved cells were seeded in the insert, placed in wells filled with 10% FBS containing medium as attractant. After 10 h incubation, the cells remained in the upper chamber were carefully removed using a cotton swab, whereas the cells that have migrated through the membrane are fixed, stained with crystal violet solution and counted.

The cell invasion rate was evaluated using the same protocol except the use of Matrigel Basement Membrane Matrix-coated modified Boyden chamber.

### Matrix Metalloproteinase activity assay

Cells were lysed in 0.1 M Tris/HCl (pH 7.5);0.1% Tween-80, on ice for 15 min, then centrifuged for 5 min at 10,000 g to discard insoluble materials. MMP-2 activity was evaluated by a colorimetric assay using Ac-Pro-Leu-Gly-[2-mercapto-4-methyl-pentanoyl]-Leu-Gly-OC2H5 thiopeptide (50 µM; BioMol International, Hamburg, Germany) in 50 mM Hepes, 10 mM CaCl_2_, 1 mM ZnCl_2_, 0.05% Brij-35, 1 mM DTNB buffer according to the manufacturer’s recommendations. The activity was expressed as treated over control ratio after correction for total protein content.

### Cell cytoplasmic labelling

The cell fluorescent labelling was performed using CytoLabeling reagent dyes (Abcam, Cambridge, UK) according to the manufacturer’s recommendations.

### Multicellular tumor spheroids

Three-dimensional (3D) culture invasion was assessed using small cell aggregates. Cells were seeded at 5 × 10^3^ cells/50 µL/well of 96-well round-bottomed plates coated with Poly-2-hydroxyethyl methacrylate (12 mg /mL; Sigma-Aldrich), and centrifuged at 200 g for 5 min to form clusters. The next day, cells were self-assembled in 3D spheroids with average diameter of 188.5 ± 8.2 µm. At day 2, the medium was supplemented with Matrigel® Basement Membrane Matrix (3.6 mg/mL) to allow cell invasion. Immunofluorescence images were acquired every 3 h over a 24 h period of time on an epifluorescence inverted microscope (Leica DM IRB, Leica Microsystems Ltd., Wetzlar Germany) equipped with a PeCon chamber i8 to perform live imaging at 37 °C with 5% CO_2_. A Leica sCMOS camera captured the dynamic process, resulting in a time-lapse movie. The microscope was steered by the Leica dedicated LasX software. Brightfield, FITC, and TRITC filter sets were used to acquire images. All quantifications were done with ImageJ software.

Larger spheroids were prepared using 5 × 10^4^ cells/50 µL/well, cultured for 10 days in complete medium, fixed in 4% paraformaldehyde then embedded in paraffin. Those larger spheroids exhibited an average diameter of 914.0 ± 173.2 µm (*n* = 30).

### Western blotting

Whole cell lysates were prepared by homogenization in buffer containing 50 mM Tris/HCl pH 7.4; 150 mM NaCl; 10 mM MgCl_2_; 1% Igepal; 10% Glycerol; 2 mM activated Na_3_VO_4_, and cocktail of protease inhibitors (Sigma Aldrich), then centrifugation at 10,000 g for 15 min to remove insoluble material. Protein concentration was determined using a colorimetric assay based on the Bradford dye-binding method (Bio-Rad Protein Assay Dye Reagent, Bio-Rad Laboratories, Marnes-la-Coquette, France). Aliquots of 30 µg proteins were separated by SDS–PAGE (sodium dodecyl sulfate–polyacrylamide gel electrophoresis), then transferred to nitrocellulose membranes (Whatman-GE Healthcare Life Sciences, Buckinghamshire, UK). The membranes were incubated for 2 h at room temperature in casein blocking buffer (Sigma Aldrich), then overnight at + 4 °C with primary antibody (Supplemental Table 3). After three washes with TBST buffer [50 mM Tris/HCl pH 7.4; 150 mM NaCl; 0.1% (v/v) Tween-20], membranes were incubated with horseradish peroxidase-conjugated secondary antibodies (1:20,000 dilution in blocking buffer) for 1 h at room temperature. After final washes, the blots were developed using enhanced chemiluminescence western blotting detection reagent (Thermo Fisher Scientific). Signals were captured using CCD camera of the ChemiDoc XRS^+^ system (BioRad Laboratories), and quantified using the ImageJ software.

### Syngeneic tumor cells graft models

Animal experiments and procedures were conducted according to the guidelines formulated by the European Commission for experimental animal use (L358-86/609EEC), and with the approval of the local ethical animal committee of Paris Saclay University (CEEA 26, Villejuif, France). In vivo assays were performed as previously described [16]. Briefly, 5-weeks old female BALB/c *nude* mice (Charles River, Arbresle, France) were randomized, and housed under pathogen-free conditions with standard mice pellet diet and water provided ad libitum. After acclimatization for 7 days, mice were injected intramuscularly with K7M2 cells (10^6^ cells/15 µL PBS) in both thighs under isoflurane/air inhalational anesthesia. Muscle infiltrated with tumor tissues and lungs were collected at one month after cell injection, fixed in 4% paraformaldehyde in PBS, and then embedded in paraffin.

### Chromogenic immunohistochemistry

Formalin-fixed paraffin-embedded (FFPE) sections (4 µm thick) dewaxed in xylene were rehydrated through a graded series of ethanol, then stained with hematoxilin-eosin or processed for IHC staining using a Bond Leica automated immuno-stainer instrument. For the IHC detection of CtBP2 and CYR61, tissue sections were processed for heat-induced antigen retrieval in citrate buffer pH6 and EDTA buffer pH9, respectively, before 1 h incubation at room temperature with primary antibody (Supplemental Table 3). Slides were then processed with the Bond Polymer Refine Detection kit (Leica Biosystems) or the Rabbit HRP PowerVision Kit (ImmunoVision Technologies). The signal was revealed with DAB, and counterstained with hematoxylin. For the evaluation of hypoxia, tissue sections were incubated for 1 h at room temperature with an anti-GLUT-1 antibody (Supplemental Table 3). The cytoplasmic signal was revealed with the Rabbit HRP PowerVision Kit (ImmunoVisionTechnologies), and with DAB completed with hematoxylin counterstaining. Whole tumor tissue sections from each sample were digitized using a slide scanner, and analyzed in a same batch. CtBP2 nucleus staining signal intensity was evaluated using QuPath software (version 0.4.2).

### Statistical analysis

GraphPad Prism Software (v10.4.1 for Windows, Boston, MA, USA; www.graphpad.com) was used to determine statistical significance between groups. *P* values < 0.05 were considered significant.

## Results

### Gene expression pattern upon CYR61 expression level

**Fig. 1:**
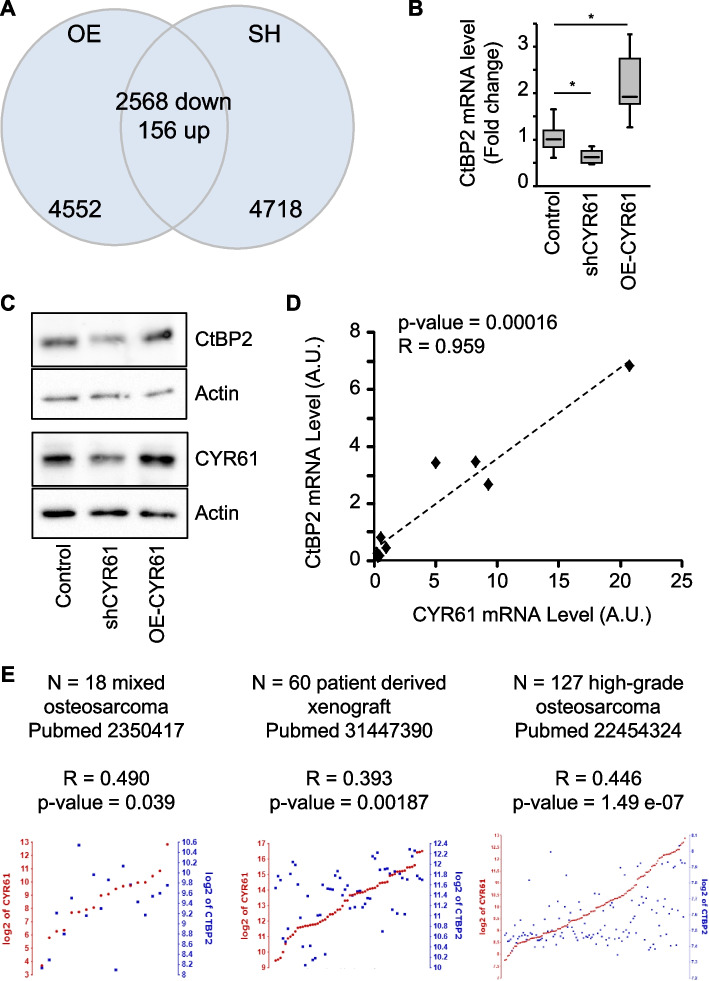
CtBP2 is a transcriptional target of CYR61. **A** Venn diagram showing the number of differentially expressed genes (DEGs) identified under Cyr61-overexpression or repression, as well as the overlap of DEGs between the two comparisons. **B** Expression pattern of CtBP2 mRNA in K7M2 cell lines modified for CYR61, as assessed by RT-qPCR. GAPDH was used as internal reference gene. The relative mRNA level was calculated using the 2^–ΔΔCT^ method and expressed as box plot (*n* = 3). An asterisk (*) indicates a statistically significant difference (*p* < 0.05). **C** Expression pattern of CtBP2 and CYR61 protein in K7M2 cell lines modified for CYR61, as assessed by western blot. Actin was used as loading control. **D** Spearman correlation between the CtBP2 and CYR61 mRNA level, evaluated by RT-qPCR in a panel of eight human osteosarcoma cell lines. The black dotted line shows the regression line. **E** Correlation plots of the CYR61 gene with the CtBP2 gene in three independent osteosarcoma sample collections revealed by the R2 platform. Spearman correlation coefficient (R) and corresponding *p*-value are reported

To investigate the modulation of gene expression depending on CYR61 levels, we performed a RNA-seq analysis of K7M2 cells stably overexpressing or silenced for CYR61 and the corresponding control cell lines. After QC confirming the high quality of the sequencing reads (Supplemental Table 4), the examination of the RNA-seq data using principal component analysis (PCA) showed a high quality of clustering between the biological replicates (Supplemental Fig. 1A). After normalization by the geometric mean of the expression levels of five housekeeping genes, volcano plots were generated to examine the distribution of log2 fold change at different significance levels. The selection criteria of q-value < 0.05 and |FC|> 15% cut-off revealed 7276 differentially expressed genes (DEGs) in CYR61-overexpressing cells, and 7442 DEGs in CYR61-silenced cells (Supplemental Figs. 1B and 1C, respectively). At the intersection of the two comparisons 2724 genes presented an opposite expression variation (Fig. 1A, Supplemental Table 5), and were considered for detailed functional analysis.

Functional interaction networks relevant to the CYR61-related set of DEGs were built using the open-source software platform Cytoscape (STRING plugin). Pathways related to “sustained proliferation signaling”, “activating invasion and metastasis”, “induced angiogenesis”, or “VEGF signaling pathway” were enriched (Supplemental Fig. 2A). These results were consistent with the pro-angiogenic and pro-metastatic functions of CYR61 we previously characterized in osteosarcoma cells [6, 16]. To explore more in depth the DEGs selection we performed enrichment analyses using the DAVID annotation tool. The reactome pathways enrichment analysis indicated that the DEGs were related to signal transduction but surprisingly also to gene expression/transcription (*p* = 0.029; Supplemental Fig. 2B). In parallel, the most significantly enriched biological process were the GO terms “transcription” and “transcription regulation” (*p* = 0.0014, and 0.0009, respectively; Supplemental Fig. 2C). Finally, we performed enrichment analyses using the Panther classification tool. Following the broad GO terms “binding” and “catalytic activity”, the GO term “transcription regulator activity” ranked the third (Supplemental Fig. 2C). This covers DNA-binding transcription factor activity and transcription co-regulator activity. Taken together, those enrichment analyses strongly suggested that CYR61 might modulate the transcription process.

Thus, we further investigated the DEGs associated with the transcription function. We identified 38 DEGs belonging to the “transcription co-regulator activity” category (GO:0003712) from the Panther analysis and the 242 DEGs belonging to the “transcription regulation” category from the DAVID analysis. We focused on the 17 DEGs in common in both classifications (Supplemental Table 6). It included genes coding mainly for transcription cofactors (35%) and chromatin-binding or -regulatory proteins and histone modifying enzymes (35%). We were interested in CtBP2 (C-terminal Binding Protein 2) as a new potential CYR61-downstream effector related to tumor cell dissemination.

### CYR61 expression level influences *CtBP2* gene expression

Quantitative RT-PCR analyses confirmed the decrease in *CtBP2* mRNA levels in CYR61-silenced K7M2 cells (−27%; *p* = 0.028; Fig. 1B) and the increase in CYR61-overexpressing K7M2 cells (+ 2.2-fold; *p* = 0.002). Western blot analyses confirmed the modulations also at a protein level (Fig. 1C). We next investigated by RT-qPCR the correlation between *CYR61* and *CtBP2* expression levels in a panel of eight human osteosarcoma cell lines. A strong positive correlation was detected between *CtBP2* and *CYR61* mRNA levels (Fig. 1D). Furthermore, a strong positive correlation was detected between *CtBP2* and *CYR61* gene expression in three independent cohorts of osteosarcoma samples from the R2 database (*p* < 0.05; Fig. 1E). We compared the survival rate in case of an alteration of *CYR61/CCN1* alone, of *CtBP2* alone or both genes, in 35 studies (39,720 samples/37,141 patients). *CYR61* alteration excluding mutation (102/3,559 analyzed patients = 2.9%) did not affect the overall survival (*p* = 0.647). In contrast, *CtBP2* alteration (86/3,559 analyzed patients = 2.4%) reduced significantly the survival rate (*p* = 0.0246; q-value = 0.0493) with a median overall survival of 15.53 *vs.* 45.04 months in unaltered group. Lastly, alteration in both *CYR61* and *CtBP2* genes also reduced the survival rate (*p* = 0.0298; q-value = 0.0596) with a median overall survival of 19.90 *vs*. 45.07 months in unaltered group.

These results suggest that the expression level of the co-transcription factor CtBP2 correlates to CYR61 expression level in the osteosarcoma cells, and may participate to the metastatic feature of osteosarcoma tumor.

To investigate the role of CtBP2 in osteosarcoma cells, we established new isogenic cell lines either repressing or over-expressing CtBP2 by cell transduction with lentiviral vectors. As expected, the stable genomic integration of *CtBP2* targeting shRNA sequences led to a reduction in *CtBP2* mRNA levels, as compared to cells transduced with non-relevant shRNA sequences (−63%, *p* = 0.002; Supplemental Fig. 3A) for K7M2 cell line. Unfortunately, the downregulation was weakly effective for U2OS cell line. Likewise, the stable genomic integration of the full length *CtBP2* coding sequence led to an increased *CtBP2* mRNA levels, as compared to control cells (2.5-fold for K7M2 cell line, *p* = 0.017 and 3.5-fold for U2OS cell lines, *p* = 0.006). Western blot analyses confirmed the modulation of CtBP2 expression at a protein level (Supplemental Fig. 3B).

### CtBP2 expression level does not influence osteosarcoma mitochondrial metabolism

**Fig. 2:**
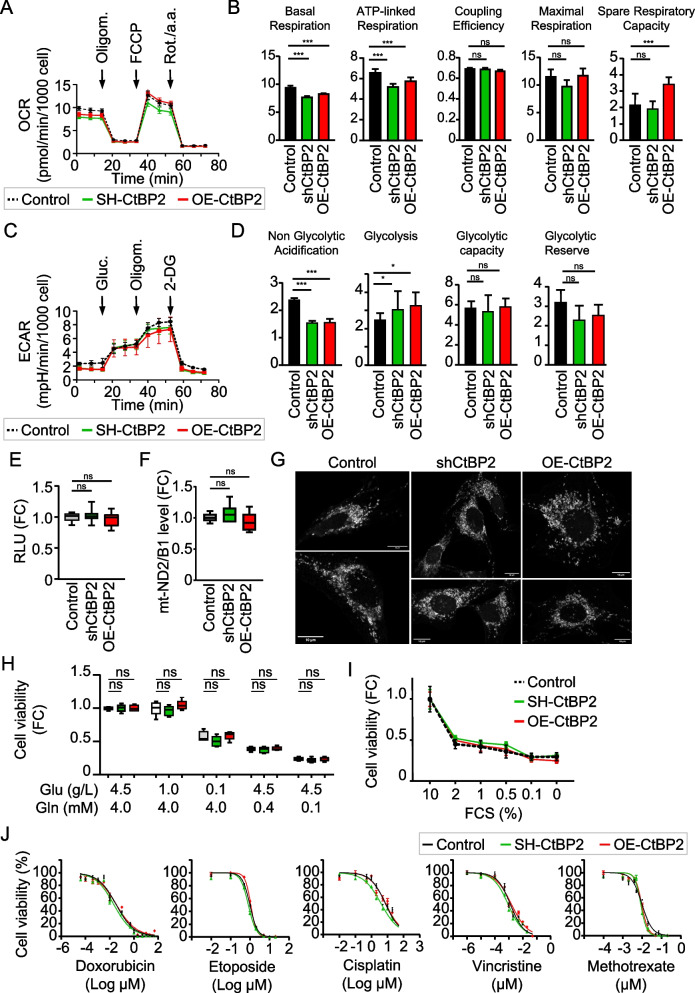
CtBP2 expression level does not affect cell sensitivity to redox stress conditions. **A** Seahorse analysis of the CtBP2-modified K7M2 cell oxygen consumption rate (OCR), normalized to cell number. **B** Relative levels of Basal respiration, ATP-liked respiration, Coupling efficiency, Maximum respiration and Spare Respiratory Capacity. Results are expressed as mean ± standard deviation (*n* = 6–8). An asterisk (*) indicates a statistically significant difference (*p* < 0.05). **C** Seahorse analysis of the extracellular acidification rate (ECAR), normalized to cell number. **D** Relative levels of non-glycolytic acidification, Glycolysis, Glycolytic capacity and Glycolytic reserve. Results are expressed as mean ± standard deviation (*n* = 6–8). An asterisk (*) indicates a statistically significant difference (*p* < 0.05). **E** Relative ATP content, as assessed by CellTiter-Glo assay. Results are expressed as mean ± standard deviation (*n* = 18). **F** Mitochondrial DNA content quantified by quantitative PCR. Results are expressed as mean ± standard deviation. **G** Mitochondrial mass and topography evaluated by confocal microscopy using Mitotracker Red staining. **H** Relative cell viability, as assessed by the MTT assay, under Glucose or Glutamine depletion for 3 days. Results are expressed as box plot (*n* = 12). **I** Relative cell viability, as assessed by the MTT assay, under FBS depletion for 3 days. Results are expressed as mean ± standard deviation (*n* = 12). **J** Relative cell viability, as assessed by the MTT assay, under treatment with the indicated chemotherapy drug for 3 days. Results are expressed as mean ± standard deviation (*n* = 12)

CtBP2 is known as a sensor of metabolic status thanks to a NAD + binding domain [33]. It is well established that tumor cells reprogram their metabolism to sustain the elevated energy demands of continuous cell growth [34]. Thus, we explored the metabolic activity of the CtBP2-modified cell lines. Using Seahorse extracellular flux analyses, we measured the real time oxygen consumption rate (OCR) and extracellular acidification rate (ECAR) to determine oxidation phosphorylation (OXPHOS) and glycolysis, respectively. Surprisingly, the measure of OCR indicated similar fluctuations for both CtBP2-silenced and overexpressing cells compared to control cells (Fig. 2A). However, even if basal and ATP-linked respiration were lower, the resulting coupling ratio of oxidation to phosphorylation (coupling efficiency) was not significantly modulated in either CtBP2-silenced or overexpressing cells (Fig. 2B). The measure of ECAR indicated similar fluctuations for both CtBP2-silenced and overexpressing cells compared to control cells: reduced non-glycolytic acidification and increased glycolysis (Fig. 2C). However, the glycolytic capacity was not significantly modulated in either CtBP2-silenced or overexpressing cells (Fig. 2D). This suggests that CtBP2 levels did not influence the ATP generation by either OXPHOS or glycolysis. Complementary experiments using CellTiter-Glo reagent confirmed that the total intracellular ATP levels were stable among the modified cell lines (Fig. 2E).

Measure of OCR following decoupling revealed Spare Respiratory Capacity (SRC) variation correlated to CtBP2 levels (Fig. 2B). Since SRC characterizes the capacity of mitochondria to meet higher energetic requests in response to cellular stress conditions, we evaluated the mitochondrial mass and structure in CtBP2-modified cells. No difference was detected between the relative mt-DNA content in the CtBP2-modified cell lines, as evaluated by qPCR (Fig. 2F). No difference were detected in the mitochondria network (morphology predominantly fragmented) or cellular location (perinuclear distribution), as evaluated by fluorescent microscopy after staining with the vital mitochondrial dye MitoTracker (Fig. 2G). Complementary experiments of cell culture under energetic substrate privation for 3 days indicated that CtBP2 levels did not influence the osteosarcoma cell response to glucose or glutamine depletion (Fig. 2H). Similar experiments under serum depletion confirmed that CtBP2 levels did not influence osteosarcoma cell sensitivity to energetic stress conditions (Fig. 2I). To further assess the response to oxidative stress, we cultured cells in the presence of increasing doses of chemotherapies. We tested doxorubicin, etoposide and cisplatin as inducer of high oxidative stress, and methotrexate and vincristine as inducer of low oxidative stress. The IC_50_ values for the five tested drugs were similar among the CtBP2-modified cell lines (Fig. 2J; Supplemental Table 7). This confirmed that CtBP2 levels did not influence osteosarcoma cell sensitivity to redox stress conditions.

### CtBP2 and CYR61 network harbors key stem factors

**Fig. 3:**
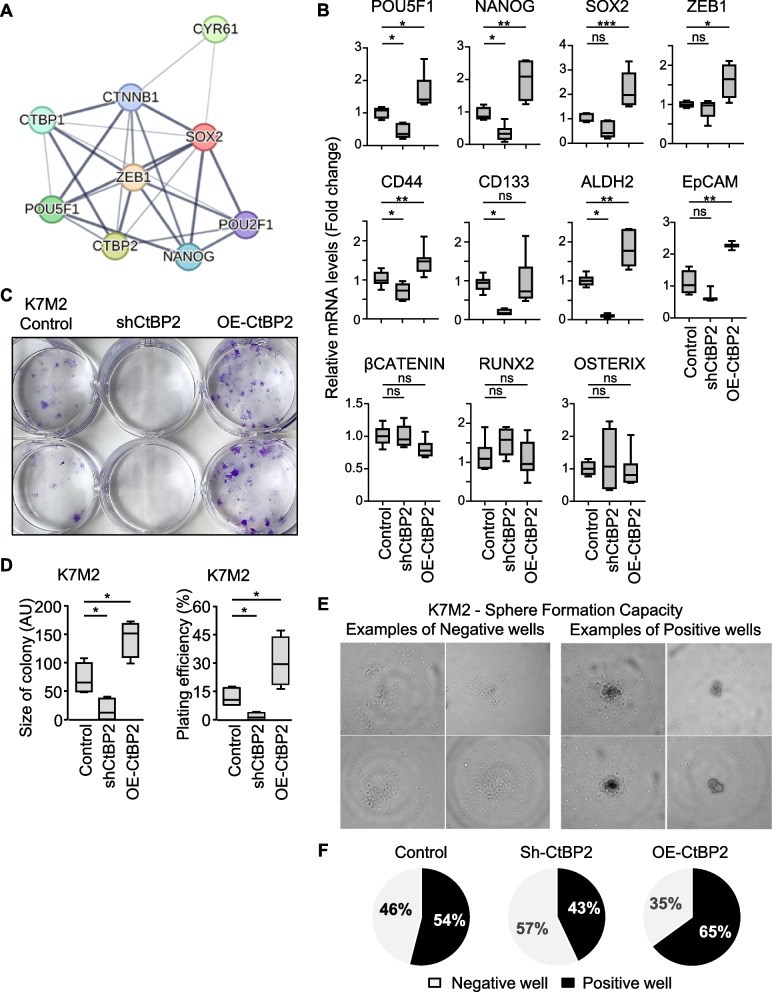
CtBP2 expression level is related to stemness. **A** Interaction network diagram of CtBP2 and CYR61 proteins. Network nodes represent proteins; the edges indicate both functional and physical protein associations; line thickness indicates the strength of data support: the thicker the gray connecting line, the stronger is the predicted interaction between the two proteins. **B** Relative mRNA levels in K7M2 modified cell lines, evaluated by RT-qPCR. GAPDH was used as internal reference gene. Results are expressed as box plot (*n* = 3–4 independent experiments). **C** Representative images from clonogenic assays after staining with crystal violet solution. K7M2 cell lines were plated at 100 cells/cm^2^, and incubated for 10 days. **D** Average size and number of colonies formed. Results are expressed as box plot (*n* = 4). An asterisk (*) indicates a statistically significant difference (*p* < 0.05 *vs.* Control). **E** Representative brightfield images of wells negative or positive for sphere formation after 10 days incubation. **F** Relative proportion of wells positive and negative for sphere formation

We next performed in silico analysis of CtBP2 and CYR61 potential protein interaction network using the STRING database. Despite the absence of direct physical or functional interaction between CtBP2 and CYR61, an indirect link was suggested through the transcription factors SOX2 (Sex determining region Y-Box 2), ZEB1 (Zinc finger E-Box binding homeobox 1), POU5F1 (POU Class 5 Homeobox 1), and NANOG Homeobox (Fig. 3A and Table 1).

**Table 1:** Interaction scores from the STRING Database as indicators of confidence in the functional or physical protein interaction. All scores rank from 0 to 1, with 1 being the highest possible confidence

	**CYR61**	**CtBP2**	**SOX2**	**ZEB1**	**POU5F1**	**NANOG**
**CYR61**	1.000					
**CtBP2**	-	1.000				
**SOX2**	0.403	0.537	1.000			
**ZEB1**	-	0.985	0.975	1.000		
**POU5F1**	-	0.700	0.999	0.679	1.000	
**NANOG**	-	-	0.998	0.960	0.999	1.000

We thus evaluated the mRNA levels of the different core stem genes in our panel of eight osteosarcoma cell lines. As expected, the expression levels of *Nanog*, *POU5F1* and *Zeb1* strongly positively correlated to each other (Table 2). We also confirmed strong positive correlation between these genes and *CtBP2* or *Cyr61* expression levels (R > 0.8).

**Table 2:** Matrix correlation of indicated mRNA levels, as assessed by RT-qPCR in a panel of eight human osteosarcoma cell lines

	**CYR61**	**CtBP2**	**SOX2**	**ZEB1**	**POU5F1**	**NANOG**
**CYR61**	1.000					
**CtBP2**	0.917	1.000				
**SOX2**	0.967	0.829	1.000			
**ZEB1**	0.959	0.847	0.934	1.000		
**POU5F1**	0.975	0.889	0.978	0.967	1.000	
**NANOG**	0.995	0.896	0.962	0.981	0.976	1.000

These results suggest that Cyr61 and CtBP2 expression correlates with the expression of stemness markers.

### CtBP2 expression level influences osteosarcoma cell stemness

We first evaluated the expression levels of stem cell markers in the CtBP2-modified cell lines. The mRNA levels of *Zeb1*, *POU5F1* and *Nanog* positively correlated to those of *CtBP2* in K7M2 cells (Fig. 3B). The mRNA levels of *CD44* and *ALDH2* also positively correlated to *CtBP2* levels, and the mRNA levels of *Sox2* and *EpCAM* were upregulated in CtBP2-overexpressing cells (Fig. 3B). In contrast, the mRNA levels of *β-catenin*, *Runx2* and *Osterix* were comparable between cell lines. Similar pattern of expression was observed in U2OS cell lines (Supplemental Fig. 3C).

These results suggest that the overexpression of CtBP2 increases osteosarcoma stemness markers, independently of osteoblastic differentiation markers.

We next performed a clonogenic assay, and observed that the ability of individual K7M2 cells to form colony was clearly dependent on CtBP2 expression levels. Indeed, the repression of *CtBP2* correlated with reduced number and size of colonies, whereas the CtBP2-overexpression promoted the colony formation ability (Fig. 3C-D). Similar pattern was observed in CtBP2-overexpressing U2OS cells (Supplemental Figs. 3D-E).

We also performed limiting dilution analysis for sphere formation, seeding 1,000 or 100 or 10 or 1 cell/well. Regardless of CtBP2 expression levels, all wells seeded with more than one cell successfully formed spheres. However, when cells were diluted at one cell/well, sphere formation capacity varied in CtBP2-dependent manner. CtBP2 silencing reduced the ability of K7M2 cells to form spheres (Fig. 3E-F), while CtBP2 overexpression increased sphere formation.

We then investigated the influence of CtBP2 levels on in vitro cell proliferation. The DNA replication rate assessed by the BrdU incorporation assay, and the relative proportions of cells in the G1, S or G2/M phases assessed by flow cytometry were similar among the CtBP2-modified cell lines (Supplemental Figs. 4A-C).

These results suggest that variations in CtBP2 expression levels influence stemness markers levels in osteosarcoma cells, without disturbance of cell proliferation.

### CtBP2 controls in vitro osteosarcoma cell migration

**Fig. 4:**
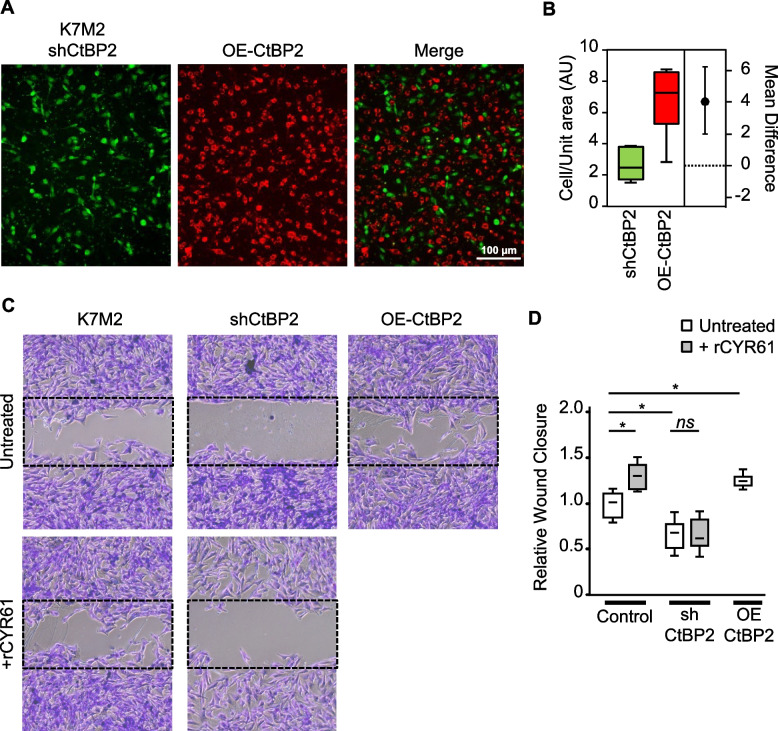
CtBP2 favors cell migration. **A** Representative images of K7M2 cell migration after 10 h incubation, as assessed by Boyden chamber assay. CtBP2-silenced K7M2 cells were stained in green, and CtBP2-overexpressing cells were stained in red before seeding (bar = 100 µm). **B** Estimation plot representing the relative number of migrating cells. The difference between the group means is represented on the right. **C** Representative images of cell migration (wound healing assay) taken at time 18 h after the wound. Control and CtBP2-silenced K7M2 cells were incubated in the presence or absence of recombinant CYR61 (1 µg/mL). The dotted rectangles outline the initial wound surface. **D** Quantitative evaluation of the cell migration rate. Results are expressed as box plot (*n* = 15–22). An asterisk (*) indicates a statistically significant difference (*p* < 0.05 *vs.* Control)

We first characterized the morphology of the CtBP2-modified cell lines by measurement of cell spreading area, perimeter, and shape/circularity (Supplemental Figs. 5A-B). All parameters were stable among the modified K7M2 and U2OS cell lines. We next assessed the migratory capacity of CtBP2-silenced and CtBP2-overexpressing cells using a modified Boyden chamber assay. After 10 h of incubation 2.5-fold more CtBP2-overexpressing cells migrated through the membrane than CtBP2-silenced cells (*p* = 0.0016; Fig. 4A-B). This ratio, showing more than twice as many migrating OE-CtBP2 cells compared to shCtBP2 cells, was validated also using modified U2OS human cell lines (*p* < 0.001; Supplemental Figs. 5C-D). A wound healing assay confirmed that CtBP2 levels modulate osteosarcoma cell motility. Indeed, the number of cells migrating into the cell-free scratch region was reduced in CtBP2-silenced population (−35%, *p* < 0.001; Fig. 4C-D), and increased in CtBP2-overexpressing population (+ 23%, *p* < 0.001), leading to an average OE/sh ratio of 1.9-fold for K7M2 cell lines. Similarly, the modified U2OS cell lines exhibited comparable behavior, with a 19% decrease (*p* = 0.0018) in CtBP2-silenced cells and a 27% increase (*p* = 0.0048) in CtBP2-overexpressing cells, resulting in an average OE/sh ratio of 1.6-fold (Supplemental Figs. 5E-F).

We next investigated the involvement of CtBP2 in the CYR61-dependent cell migration process we previously described [6, 16, 17, 35]. As expected, the supplementation of culture medium with recombinant CYR61 increased the number of migrating K7M2 cells (+ 30%, *p* = 0.025 *vs.* untreated; Fig. 4C-D). Interestingly, recombinant CYR61 supplementation did not significantly modify CtBP2-silenced cell migration rate (*p* = 0.46). Correspondingly, supplementation with recombinant CYR61 increased the number of migrating U2OS cells (+ 28%, *p* = 0.0026; Supplemental Figs. 5E-F), but not of CtBP2-silenced cells (*p* = 0.19).

Taken together, these results suggest that CtBP2 upregulation supports osteosarcoma cell migration. Moreover, these results indicate that osteosarcoma cell motility requires minimal CtBP2 expression levels and that CYR61-mediated cell migration is at least in part dependent on CtBP2 upregulation.

### CtBP2 controls in vitro osteosarcoma cell invasiveness

**Fig. 5:**
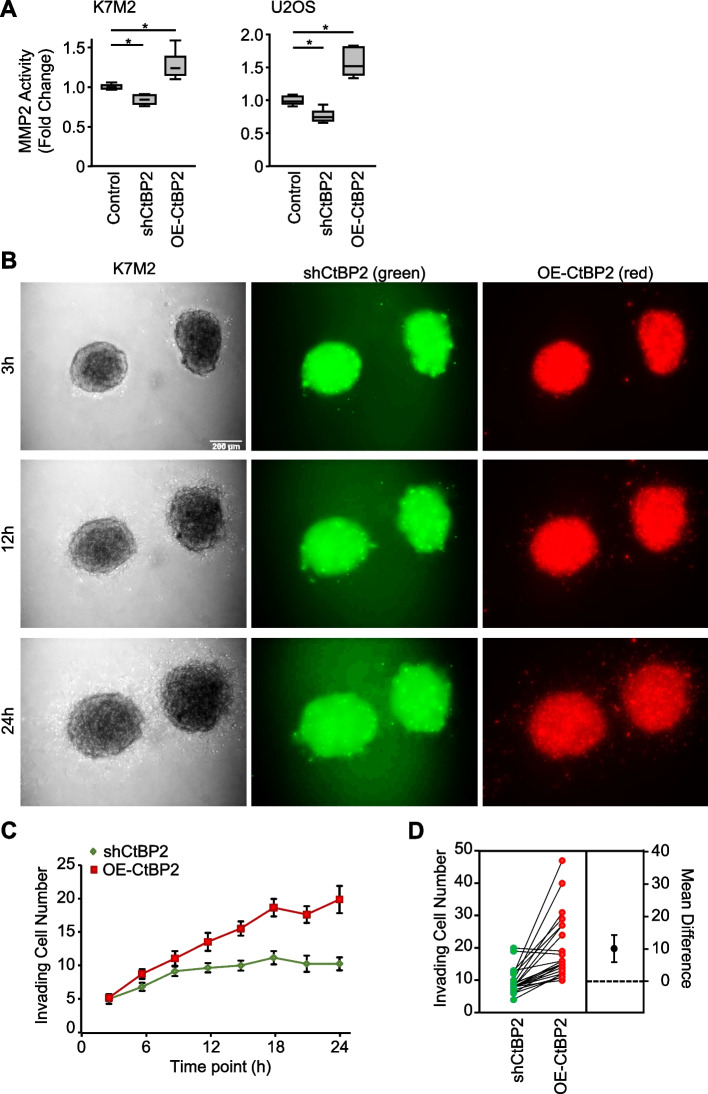
CtBP2 favors cell invasion through extracellular matrix. **A** Relative Matrix Metalloproteinase-2 (MMP2) activity in K7M2 and U2OS modified cell lines, as assessed by colorimetric assay. Results are expressed as box plot (*n* = 10). An asterisk (*) indicates a statistically significant difference (*p* < 0.05 *vs.* Control). **B** Representative images of spatial spheroid invasion assays. CtBP2-silenced K7M2 cells were stained in green, and CtBP2-overexpressing cells were stained in red (bar = 200 µm). **C** Quantitative evaluation of the cells invading the Matrigel basement membrane at different time points. Results are expressed as mean ± standard error of the mean (*n* = 23 scored spheroids from three independent experiments).** D** Estimation plot representing the relative number of invading cells as compared to Control (paired per spheroid). The difference between the group means is represented on the right

Next, we examined the impact of CtBP2 expression levels on cell invasiveness. First, we evaluated the MMP-2 activity that plays an important role in the invasive process of osteosarcoma cells [3, 4, 6, 17]. CtBP2-silenced K7M2 cells exhibited a reduced MMP-2 activity compared to Control cells (−17%, *p* = 0.0012; Fig. 5A), whereas CtBP2-overexpressing cells exhibited a higher MMP-2 activity (+ 27%, *p* = 0.0083). In the same manner, CtBP2-silenced U2OS cells exhibited a reduced MMP-2 activity compared to control cells (−24%, *p* = 0.0016), whereas CtBP2-overexpressing cells exhibited higher MMP-2 activity (+ 57%, *p* < 0.0001).

Second, we investigated the ability of the modified cell lines to invade an extracellular matrix. 3D condensed structures (spheroids) established by homogeneously mixing pre-stained CtBP2-silenced and -overexpressing cells (1:1 ratio) were incubated in the presence of Matrigel basement membrane. Cells penetrating the surrounding matrix environment were monitored and scored at different time points (Fig. 5B-C). CtBP2-overexpressing cells that invade the Matrigel are more numerous than CtBP2-silenced cells, representing about two third of invading cells after 24 h (*p* < 0.0001; Fig. 5C-D).

These results show that CtBP2 overexpression promotes Matrix Metalloproteinase activity that leads to osteosarcoma cell invasion.

### Overexpression of CtBP2 at the invasive front of the tumors

**Fig. 6:**
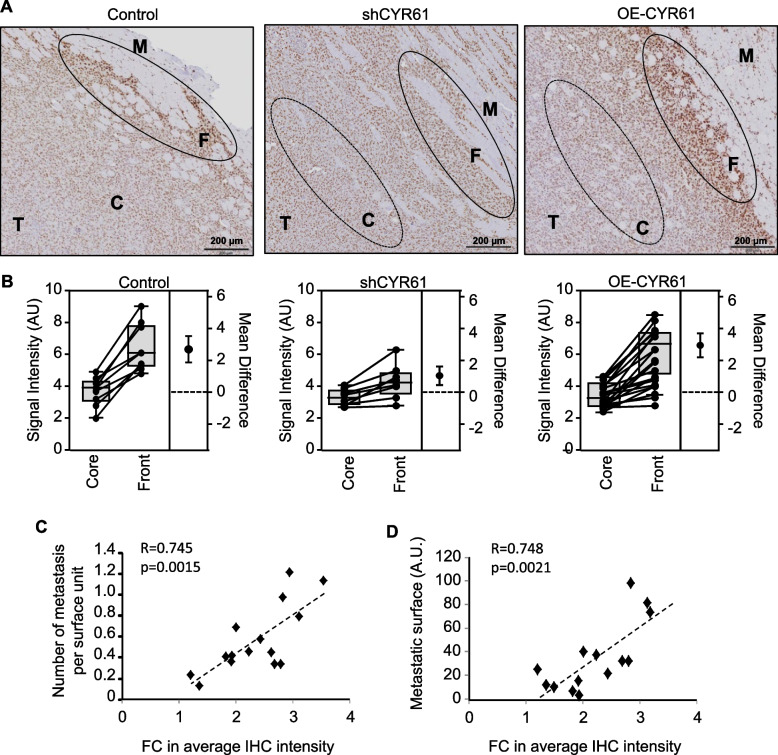
CtBP2 induction by CYR61 is maximal at the invasive front of the tumor. **A** CtBP2 immunohistochemical staining of FFPE tissue sections of Control, CYR61-silenced or CYR61-overexpressing cell line derived xenografts. Tumor (T) and surrounding muscle (M) as well as the tumor core area (C) and invasive front (F) are indicated. The scale bars represent 200 µm. **B** Estimation plots representing the relative CtBP2 signal intensity determined for Control, CYR61-silenced or CYR61-overexpressing cell line derived xenografts. Results are expressed as box plot and paired individual sample comparison between the tumor core and invasive front. The difference between the means are represented on the right of each plot. **C** Spearman correlation between the average CtBP2 signal intensity and the number of metastatic foci in the lungs. The dotted line shows the regression line. **D** Spearman correlation between the average CtBP2 signal intensity and the lung metastatic surface. The dotted line shows the regression line

To confirm the role of CtBP2 in tumor dissemination we used syngeneic tumor mouse models. We implanted K7M2 cells into tight muscle of BALB/c-*nu* mice to generate primary osteosarcoma tumors. IHC staining on FFPE sections of Control cells-derived tumors revealed a heterogeneity in the CtBP2 signal intensity within the tissue section (Fig. 6A). Interestingly, osteosarcoma cells invading the surrounding normal tissue exhibited a higher signal intensity for CtBP2 as compared to cells located in a more core area (+ 79%; *p* = 0.0002; Fig. 6B). We thereafter investigated CtBP2 signal intensity in FFPE sections of tumor samples derived from the CYR61-modified cells. Surprisingly, no significant difference was detectable in CtBP2 signal intensity at the core area of tissue sections between the different models (*p* > 0.3). Nevertheless, a similar profile to the Control group was detected for the CYR61-overexpressing model, with an increase in CtBP2 signal intensity at the invasive front as compared to the core area (+ 84%; *p* = 0.0002; Fig. 6A-B, Supplemental Fig. 6A). In contrast, CtBP2 signal in cells located at the front area of the tumor derived from CYR61-silenced grafted cells was weakly increased as compared to the core area (+ 29%; *p* = 0.0178).

We also assessed the correlation between variations in CtBP2 expression levels and the incidence of metastases. We observed a strong positive correlation between the upregulation of CtBP2 at the invasive tumor front and both the number and the surface area of pulmonary metastatic foci (Fig. 6C-D; Supplemental Fig. 6B).

These results suggest that CtBP2 expression in CYR61-expressing osteosarcoma cells is induced at the invasive front and correlates with the metastatic dissemination process. In contrast, in the central region of the tumors, there is a distinct blockade of the CYR61-dependent induction of CtBP2 expression.

### Micro-environmental conditions impair CtBP2 induction

**Fig. 7:**
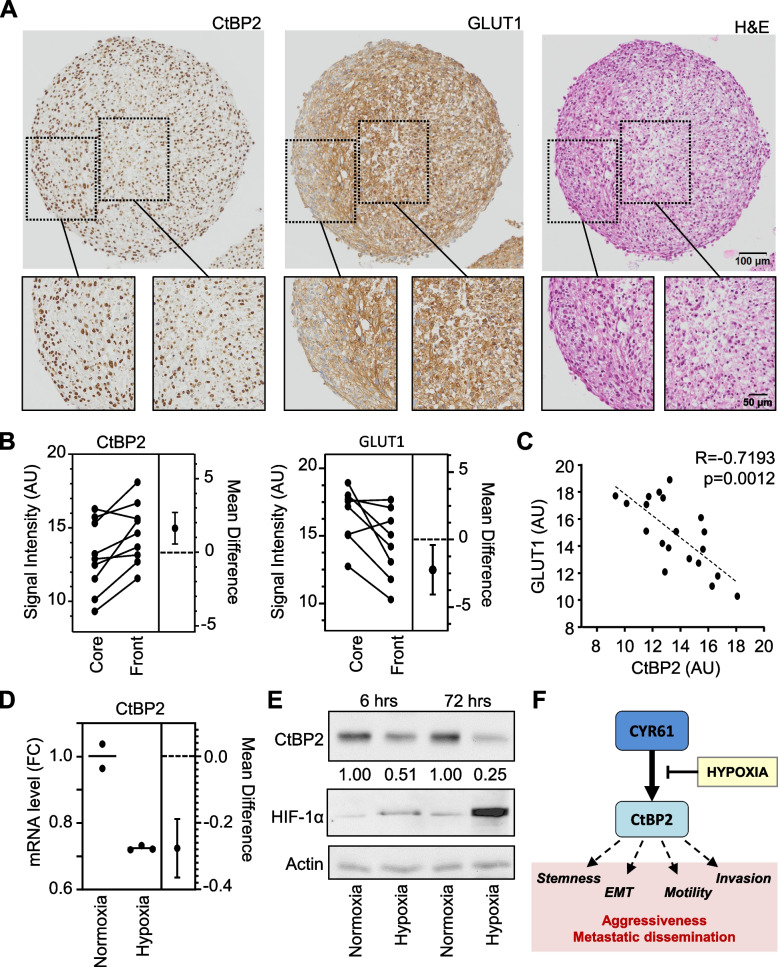
CtBP2 expression is dependent on oxygen levels. **A** Immunohistochemical staining for CtBP2 and GLUT1, and Hematoxylin and Eosin staining of FFPE section of K7M2 cultured as 3D spheroid. The scale bars represent 100 µm or 50 µm for below insert. **B** Estimation plots representing the relative CtBP2 and GLUT1 signal intensity. Results are expressed as paired sample comparison between the spheroid core and front area (*n* = 10). The difference between the means are represented on the right of each plot. **C** Spearman correlation between CtBP2 and GLUT1 signal intensities. The dotted line shows the regression line. **D** Estimation plot representing the CtBP2 mRNA level variation in K7M2 cell line cultured under normoxic or hypoxic conditions, as assessed by RT-qPCR. The difference between the group means is represented on the right. **E** Expression pattern of CtBP2 and HIF-1α protein in K7M2 cells cultured as 2D monolayer for 6 h or 72 h in normoxic or hypoxic condition, as assessed by western blot. Actin was used as loading control. **F** Proposed model summarizing our data. CtBP2 is a transcriptional target of CYR61. Hypoxia prevents this. CtBP2 triggers EMT, acquisition of stem cell-like characteristics, higher cell motility and invasiveness, which all together promote aggressiveness and osteosarcoma cell dissemination

To validate the role of spatial location within the tumor mass in the relationship between CYR61 and CtBP2 expression levels, we established larger 3D spheroids. As expected, we observed a gradient in CtBP2 signal intensity, with higher levels in the cells at the outer proliferating edge of the spheroid FFPE sections as compared to the core (average + 10%, *p* = 0.022; Fig. 7A-B). Since the core of large spheroids usually lacks nutrients and oxygen, we evaluated the expression levels of GLUT1 as marker to detect hypoxia. We observed a gradient in signal intensity with lower expression level in the cells at the outer edge of the spheroid as compared to those in the core (average −8%, *p* = 0.029). Overall, we observed a strong negative correlation between the signal intensities of CtBP2 and GLUT1 (Fig. 7C). To test if oxygen levels influence CtBP2 expression, we incubated K7M2 cells as 2D monolayers under either normoxic or hypoxic conditions (3% oxygen). As expected, we observed a marked decrease in CtBP2 expression levels in cells cultured under hypoxia as compared to normoxia at both mRNA and protein levels (Fig. 7D-E).

These results suggest that hypoxic conditions impair CtBP2 induction of expression, while non-hypoxic conditions such as the external location of tumor cell aggregates promote its expression.

## Discussion

One of the most significant negative prognostic factor for survival of osteosarcoma patients is the presence of metastasis at the time of diagnosis [36]. Thus, preventing the metastatic dissemination is a crucial issue for treatment success. Others and we have previously demonstrated that CYR61 is a key pro-metastatic factor in bone tumor cells [6, 16, 17]. CYR61 is a secreted signaling protein associated with extracellular matrix (ECM) that interacts with several membrane receptors (reviewed in [37]). The resulting signaling cascades activate various intracellular effectors such as PI3K-Akt / ERK1/2-MAPKs / NFkB / ILK / TEAD-YAP, and lead to the transcriptional activation of genes related to proliferation, migration, invasion and angiogenesis.

Among the transcription cofactors, LIMD1 (LIM Domain Containing 1) and AJUBA LIM Protein are associated with the activation of YAP/TAZ (Yes-Associated Protein/Transcriptional co-Activator with PDZ‐binding motif) [38, 39]. This finding aligns with the literature that connects CYR61 to TEAD proteins-related signaling [40]. Transcriptomic dataset analyses predicted PPRC1 (Peroxisome Proliferator-Activated Receptor Gamma Coactivator-Related Protein 1) to be engaged in the regulation of actin polymerization under type V collagen overexpression, favoring cell mobility and metastatic process in glioblastoma [41]. TCERG1 (Transcription Elongation Regulator 1) binds RNA polymerase II and inhibits the elongation of transcripts from target promoters. In hepatocellular carcinoma, high TCERG1 expression associates with enhanced cell cycle, migration and invasion, leading to a poor prognosis [42]. The CtBP family of proteins serve as transcriptional co-regulators implicated in mammalian embryogenesis and development, and oncogenesis (reviewed in [43]). The regulatory activity depends on their interaction with various partner proteins [44]. Thus, CtBPs exhibit a predominant co-repressor activity thereby contributing to the negative regulation of the expression of many tumor suppressor genes (reviewed in [45]). In addition, CtBPs can also exhibit co-activating activity favoring the expression of genes that promote proliferation and cancer stem cell self-renewal, or EMT [46], as we have previously reported the induction of an EMT-like process in osteosarcoma cells overexpressing CYR61 [17]. In silico analysis suggested that the CYR61 and CtBP2 interaction network may involve stemness markers, namely SOX2, NANOG, ZEB1 and POU5F1. We confirmed a strong positive correlation (R > 0.8) between CYR61, CtBP2 and the stemness markers expression at the transcriptional levels in different osteosarcoma cell lines. Moreover, our functional validation experiments suggest that CtBP2 favors the acquisition of stem cell-like characteristics. These results strengthen our hypothesis that CtBP2 plays a crucial role in the CYR61-dependent induction of EMT-like process of osteosarcoma cells and is required for tumor cell dissemination. To our knowledge, no existing data links CtBP2 and CYR61/CCN1 in any type of tumor or normal cells.

The genomic chaos characterizing osteosarcoma cells drives the heterogeneity and complexity of this cancer. The random but extensive DNA rearrangements, named chromothripsis [47], are a crucial issue halting the development of effective therapies to complement chemotherapy that are frequently inefficient. In this context, the expression of genes related to DNA repair was reported to be controlled by a transcriptional complex comprising CtBP1/2 heterodimer, CtBP-interacting protein (CtIP), histone deacetylase 1 (HDAC1), and two subunits of activating protein 1 (AP1) [48].

Another critical concern is the metastatic spread of cancer to distant organs. An increased expression of CtBP2, but not CtBP1, was reported in a small cohort of tumor tissue samples (*n* = 28) compared to noncancerous bone tissue samples [49]. The overexpression of CtBP2 also positively associated with the presence of pulmonary metastasis, and worse survival rate. In the present study, we observed a positive correlation between CtBP2 levels in osteosarcoma cells and their motility in vitro as well as their invasiveness in vivo. Surprisingly, CtBP2 levels did not affect either energetic mitochondrial metabolism or mitochondrial network structure and mass. In addition, CtBP2 levels did not influence the use of nutrients (*i.e.* glucose, glutamine, FBS) nor the sensitivity to chemotherapeutic agents delineating a metabolism-independent mechanism, at least in vitro. We also provide evidence that CtBP2-silencing lowered the migration capacity in vitro in 2D and 3D models, and prevented CYR61-dependent induction of cell migration. Moreover, we reported that CtBP2-silencing significantly impairs tumor dissemination capacity in vivo.

Overall, we identified CtBP2 as a downstream transcriptional target of CYR61, and demonstrated that CtBP2 expression is required for CYR61-dependent pro-metastatic dissemination of osteosarcoma, by favoring cell migration and invasiveness. Interestingly, we observed that CtBP2 repression haltered the pro-metastatic cascade initiated by CYR61. CtBP2 thus represents an interesting potential target for the prevention of tumor spreading. This observation could not be restricted just to osteosarcoma as CtBPs hyperactivity mediates oncogenic functions and promotes aggressive and metastatic neoplastic behavior in various solid tumors (reviewed in [50]). Attempts to test compounds that interfere with CtBP protein–protein interactions or dehydrogenase enzymatic activity in in vivo assays have shown limited success [50]. The development of derivative or related molecules is on the way, and such inhibitors could represent a valuable treatment option for osteosarcoma.

Another key finding from our study is that the spatial location of tumor cells within the tumor mass is critical for the good execution of the CYR61-CtBP2-migration/invasion cascade. Indeed, the highest levels of CtBP2 expression was detectable in the surrounding cells whereas no upregulation of CtBP2 was detectable in the cells located in a more central area. The presence of hypoxia responsive elements in the CtBP2 promoter [51] suggests that HIF family members interfere with the induction of CtBP2 expression. In fact, CtBP has been described as critical mediator of the oxygen-sensing response [52, 53]. Our in vitro experiments confirmed the role of micro-environmental conditions such as the oxygen levels in the regulation of CYR61-dependent induction of CtBP2 expression by osteosarcoma cells.

## Conclusions

Clinical management of osteosarcoma requires new therapeutic strategies to prevent the development of metastatic disease, and thus improve the outcomes for patients with poor prognosis. Our previous studies, along with research by others, strongly suggest CYR61/CCN1 as a prognosis biomarker of metastatic dissemination. The present study provides new information on the CYR61-driven pro-metastatic cascade, involving the transcriptional induction of the co-repressor CtBP2 (Fig. 7F). Our data also show that this cascade occurs under oxygen-driven conditions, as encountered rather at a peripheral location of the tumor, representing the invasive front. CtBP2 is indeed required for pro-metastatic dissemination of osteosarcoma, by favoring cell migration and invasiveness. Hence, CtBP2 is a crucial element of the CYR61-driven invasiveness of osteosarcoma cells.

## Supplementary Information

Additional file 1: Supplemental Figure 1. A Principal Component Analysis (PCA) of gene expression data obtained via normalized RNA-seq read counts for five biological replicates corresponding to the samples from K7M2 cells stably overexpressing (green dots) or silenced (red dots) for CYR61 and the corresponding control cell lines (blue and brown dots, respectively). Sample to sample distances (within-and between-groups) are illustrated on the first two principal components space, based on the 1000 more contributing genes. B Volcano plot illustrating the gene expression levels variation between Cyr61-overexpressing and parental cells. Down- and upregulated genes (|FC| ≥ 15% and q-value <0.05) are reported as blue dots, whereas the black dots represent insignificant DEGs. C Volcano plot illustrating the gene expression levels variation between Cyr61-repressed and control cells. Down- and upregulated genes (|FC| ≥ 15% and q-value <0.05) are reported as blue dots, whereas the black dots represent insignificant DEGs. Supplemental Figure 2. A Functional interaction networks from Cytoscape open source software platform. Curated and experimentally derived pathways with the highest number of query genes overlapping the network set are listed. B Reactome pathways and gene ontology enrichment analyses of DEGs using DAVID annotation tool. The five most significant GO terms for reactome pathways (blue) and biological process (red) are illustrated. C Distribution of gene ontology molecular functions corresponding to the identified DEGs, established using PANTHER classification system. The five highest classes are detailed. Supplemental Figure 3. A Expression pattern of CtBP2 mRNA in Control and stably modified cell lines, as assessed by RT-qPCR. GAPDH was used as internal reference gene. The relative mRNA level was calculated using the 2^–ΔΔCT^ method and expressed as box plot (*n*=3 independent experiments). An asterisk (*) indicates a statistically significant difference (*p*<0.05 *vs.* Control). B Expression pattern of CtBP2 protein in K7M2 and U2OS modified cell lines, as assessed by western blot. Actin was used as loading control. C Relative mRNA levels in U2OS modified cell lines, evaluated by RT-qPCR. GAPDH was used as internal reference gene. Results are expressed as box plot (*n*=3 independent experiments). D Representative images from clonogenic assays after staining with crystal violet solution. U2OS cell lines were plated at 100 cells/cm², and incubated for 10 days. E Average size and number of colonies formed. Results are expressed as box plot (*n*=4). An asterisk (*) indicates a statistically significant difference (*p*<0.05 *vs.* Control). Supplemental Figure 4. A Relative DNA replication rate, as assessed by the BrdU incorporation assay. Results are expressed as mean ± standard deviation (*n*=17). B Relative proportion of cells in G1, S or G2 phase of cell cycle, evaluated by flow cytometry. Results are expressed as mean ± CV. C Flow cytometry analysis for cell cycle distribution. Supplemental Figure 5. A Representative images of K7M2 and U2OS modified cell morphology. B Distribution of cell shape parameters (cell surface, cell perimeter, and circularity index) for the Control and stably modified K7M2 and U2OS cells. Results are expressed as box plot (*n*=105-130). C Representative images of U2OS cell migration after 10 h incubation, as assessed by Boyden chamber assay. CtBP2-silenced U2OS cells were stained in green, and CtBP2-overexpressing cells were stained in red (bar= 100 µm). D Estimation plot representing the relative number of migrating cells as compared to Control. The difference between the group means is represented on the right. E Representative images of cell migration (wound healing assay) taken at time 18h after the wound. Control and CtBP2-silenced U2OS cells were incubated in the presence or absence of recombinant CYR61 (1 µg/mL). The dotted rectangles outline the initial wound surface. F Quantitative evaluation of the cell migration rate. Results are expressed as box plot (*n*=6). An asterisk (*) indicates a statistically significant difference (*p*<0.05 *vs.* control). Supplemental Figure 6. A Representative images with higher magnification of CtBP2 immunohistochemical staining on FFPE tissue sections of CYR61-silenced or CYR61-overexpressing cell line derived xenografts. Tumor (T) and surrounding muscle (M) as well as the tumor core area (C) and invasive front (F) are indicated. B Representative images of HE staining of lung tissue sections. Arrows indicate examples of metastatic foci.

Additional file 2

## Data Availability

The RNA-seq raw data generated for this study are available at the European Genome-phenome Archive (EGA) under the accession number EGAD50000000747.

## Abbreviations

BrdU: Bromo-deoxyuridine
CtBP2: C-terminal Binding Protein-2
CTGF: Connective Tissue Growth Factor
CYR61: Cysteine Rich Protein-61
DAPI: 4’,6-Diamidino-2-phenylindole
DEGs: Differentially Expressed Genes
DTNB: 5,5’-Dithiobis-(2-Nitrobenzoic Acid)
ECAR: Extracellular acidification rate
EFS: Event Free Survival
EMT: Epithelial to Mesenchymal Transition
FFPE: Formaldehyde Fixed Paraffin Embedded
GAPDH: Glyceraldehyde-3-Phosphate Dehydrogenase
GLUT1: Glucose Transporter 1
GSEA: Gene Set Enrichment Analysis
H&E: Hematoxylin and Eosin
HIF-1α: Hypoxia Inducible Factor 1 Subunit Alpha
IHC: Immuno-Histochemistry
MMP: Matrix Metalloproteinase
mt-DNA: Mitochondrial DNA
MTT: 3-(4,5-Dimethylthiazol-2-yl)-2,5-diphenyltetrazolium bromide
NANOG: Homeobox Transcription Factor Nanog
NOV: Nephroblastoma Overexpressed
OCR: Oxygen consumption rate
OS: Overall Survival
PCA: Principal Component Analysis
POU5F1: POU class 5 Homeobox 1
RNA-seq: Ribonucleic acid sequencing
RT-qPCR: Reverse Transcription-quantitative Polymerase Chain Reaction
SOX2: SRY (Sex determining Region Y)-Box 2
SRC: Spare Respiratory Capacity
WISP: WNT1-Inducible-Signaling Pathway
ZEB1: Zinc finger E-Box binding homeobox 1
