# Supplementary material for: C-terminal binding protein-2 triggers CYR61-induced metastatic dissemination of osteosarcoma in a non-hypoxic microenvironment
Source: J Exp Clin Cancer Res. 2025 Mar 5;44:83. doi: 10.1186/s13046-025-03350-6 (PMC11881356; doi:10.1186/s13046-025-03350-6)
Supplement: Supplementary file 1 — Additional file 1: Supplemental Figure 1. A Principal Component Analysis (PCA) of gene expression data obtained via normalized RNA-seq read counts for five biological replicates corresponding to the samples from K7M2 cells stably overexpressing (green dots) or silenced (red dots) for CYR61 and the corresponding control cell lines (blue and brown dots, respectively). Sample to sample distances (within-and between-groups) are illustrated on the first two principal components space, based on the 1000 more contributing genes. B Volcano plot illustrating the gene expression levels variation between Cyr61-overexpressing and parental cells. Down- and upregulated genes (|FC| ≥ 15% and q-value <0.05) are reported as blue dots, whereas the black dots represent insignificant DEGs. C Volcano plot illustrating the gene expression levels variation between Cyr61-repressed and control cells. Down- and upregulated genes (|FC| ≥ 15% and q-value <0.05) are reported as blue dots, whereas the black dots represent insignificant DEGs. Supplemental Figure 2. A Functional interaction networks from Cytoscape open source software platform. Curated and experimentally derived pathways with the highest number of query genes overlapping the network set are listed. B Reactome pathways and gene ontology enrichment analyses of DEGs using DAVID annotation tool. The five most significant GO terms for reactome pathways (blue) and biological process (red) are illustrated. C Distribution of gene ontology molecular functions corresponding to the identified DEGs, established using PANTHER classification system. The five highest classes are detailed. Supplemental Figure 3. A Expression pattern of CtBP2 mRNA in Control and stably modified cell lines, as assessed by RT-qPCR. GAPDH was used as internal reference gene. The relative mRNA level was calculated using the 2–ΔΔCT method and expressed as box plot (n=3 independent experiments). An asterisk (*) indicates a statistically significant difference (p<0.05 vs [file 13046_2025_3350_MOESM1_ESM.pptx]

## Slide 1
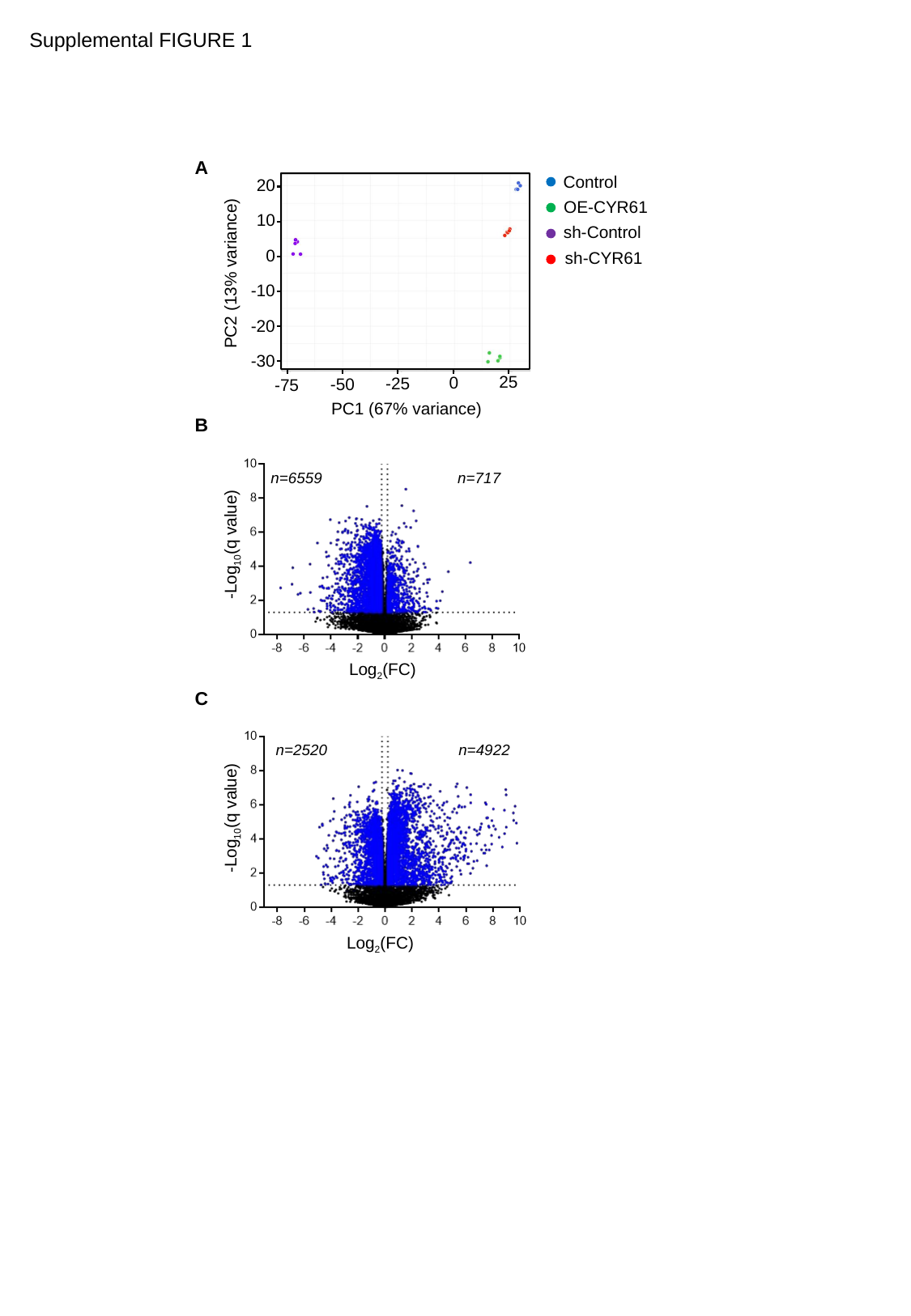

Supplemental FIGURE 1
A
Control
OE-CYR61
sh-Control
sh-CYR61
20
10
0
PC2 (13% variance)
-10
-20
-30
25
0
-25
-50
-75
PC1 (67% variance)
B
n=6559
n=717
-Log10(q value)
Log2(FC)
C
n=2520
n=4922
-Log10(q value)
Log2(FC)

## Slide 2
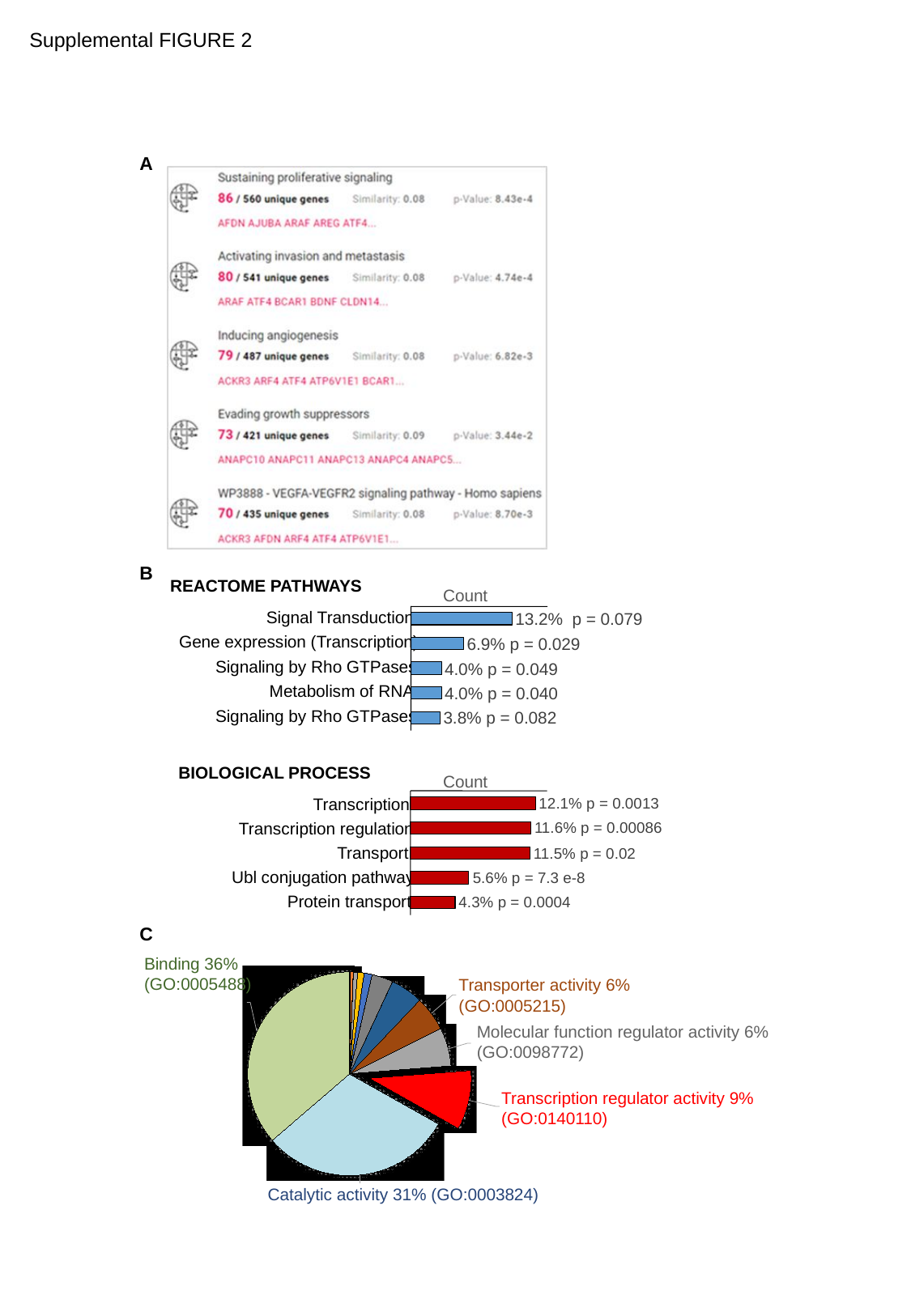

Supplemental FIGURE 2
A
B
REACTOME PATHWAYS
Count
Signal Transduction
13.2% p = 0.079
Gene expression (Transcription)
6.9% p = 0.029
Signaling by Rho GTPases
4.0% p = 0.049
Metabolism of RNA
4.0% p = 0.040
Signaling by Rho GTPases
3.8% p = 0.082
BIOLOGICAL PROCESS
Count
Transcription
12.1% p = 0.0013
Transcription regulation
11.6% p = 0.00086
Transport
11.5% p = 0.02
Ubl conjugation pathway
5.6% p = 7.3 e-8
Protein transport
4.3% p = 0.0004
C
Binding 36%
(GO:0005488)
Transporter activity 6%
(GO:0005215)
Molecular function regulator activity 6%
(GO:0098772)
Transcription regulator activity 9%
(GO:0140110)
Catalytic activity 31% (GO:0003824)

## Slide 3
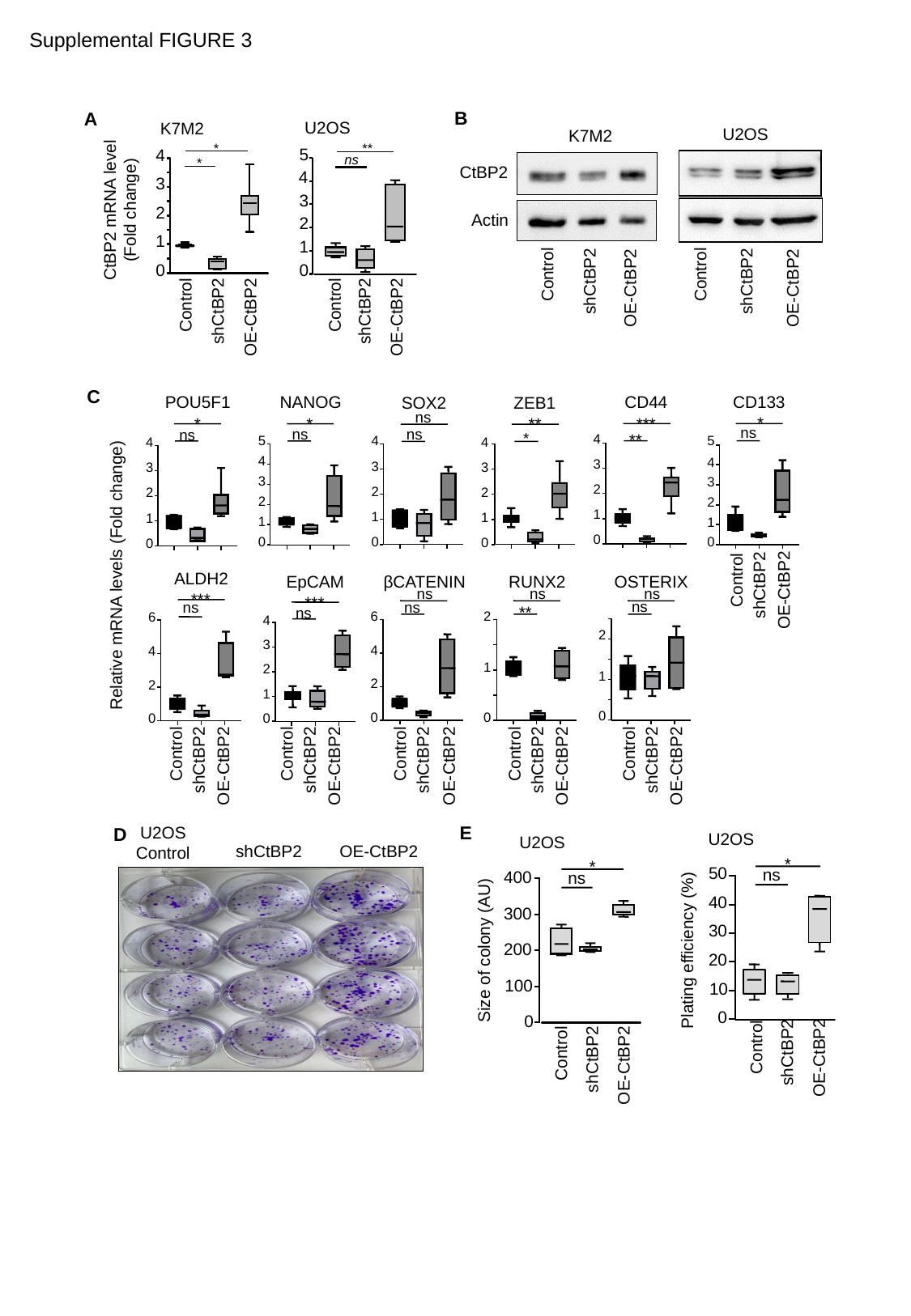

Supplemental FIGURE 3
B
A
U2OS
**
5
ns
4
3
2
1
0
Control
shCtBP2
OE-CtBP2
K7M2
*
4
3
2
1
0
*
Control
shCtBP2
OE-CtBP2
U2OS
K7M2
CtBP2
Actin
Control
Control
shCtBP2
shCtBP2
OE-CtBP2
OE-CtBP2
CtBP2 mRNA level
(Fold change)
C
CD44
CD133
POU5F1
NANOG
SOX2
ZEB1
ns
*
***
*
*
**
ns
ns
ns
ns
*
**
4
5
4
5
4
4
4
4
3
3
3
3
3
3
2
2
2
2
2
2
1
1
1
1
1
1
0
0
0
0
0
0
Relative mRNA levels (Fold change)
ALDH2
Control
EpCAM
βCATENIN
RUNX2
OSTERIX
shCtBP2
OE-CtBP2
ns
ns
ns
***
***
ns
ns
ns
**
ns
2
6
6
4
2
3
4
4
1
2
1
2
2
1
0
0
0
0
0
Control
Control
Control
Control
Control
shCtBP2
shCtBP2
shCtBP2
shCtBP2
shCtBP2
OE-CtBP2
OE-CtBP2
OE-CtBP2
OE-CtBP2
OE-CtBP2
E
U2OS
Control
shCtBP2
OE-CtBP2
D
U2OS
*
50
ns
40
30
Plating efficiency (%)
20
10
0
Control
shCtBP2
OE-CtBP2
U2OS
*
400
ns
300
200
Size of colony (AU)
100
0
Control
shCtBP2
OE-CtBP2

## Slide 4
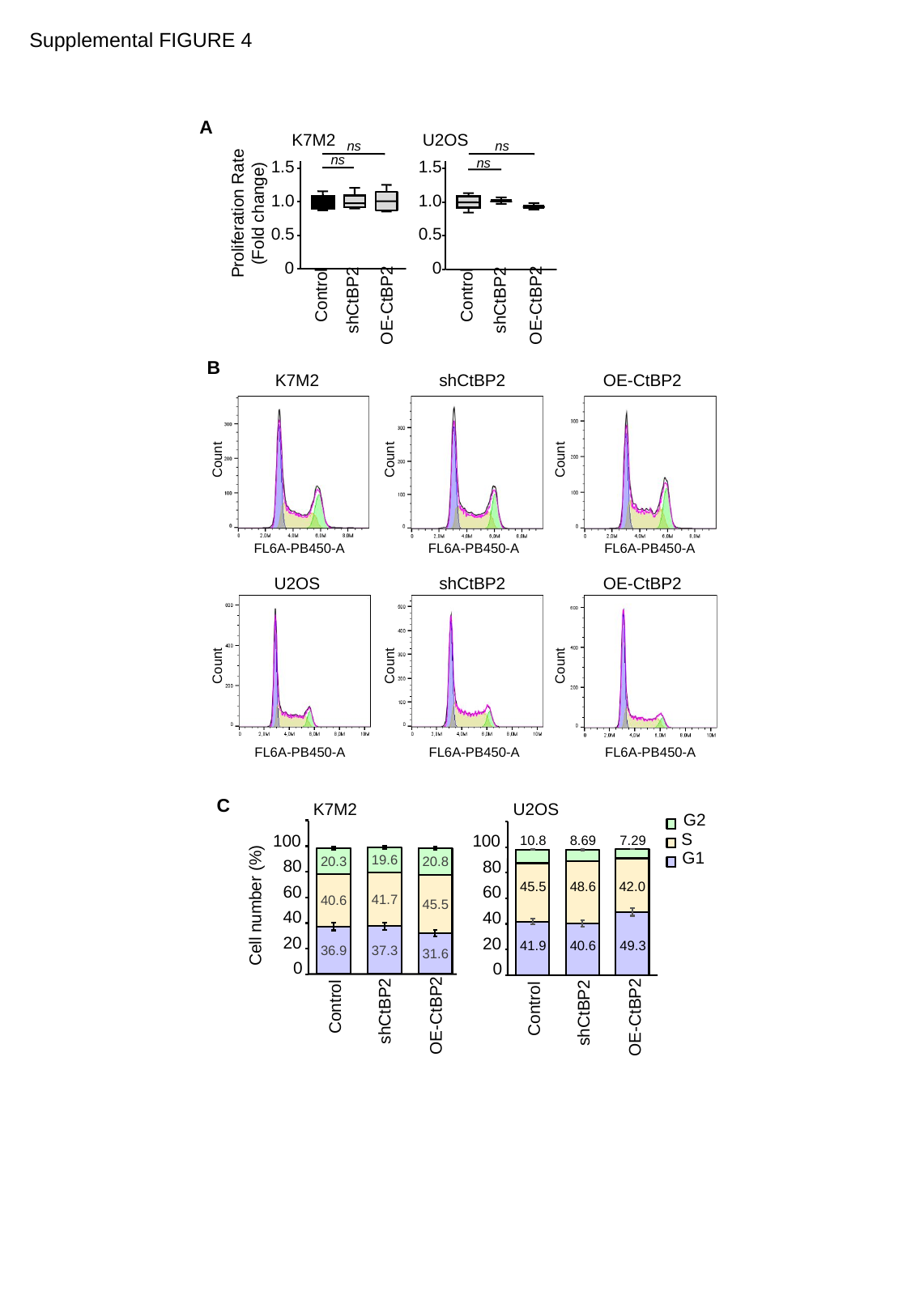

Supplemental FIGURE 4
A
U2OS
K7M2
ns
ns
ns
ns
1.5
1.5
1.0
1.0
Proliferation Rate
(Fold change)
0.5
0.5
0
0
Control
Control
shCtBP2
shCtBP2
OE-CtBP2
OE-CtBP2
B
K7M2
shCtBP2
OE-CtBP2
Count
Count
Count
FL6A-PB450-A
FL6A-PB450-A
FL6A-PB450-A
U2OS
shCtBP2
OE-CtBP2
Count
Count
Count
FL6A-PB450-A
FL6A-PB450-A
FL6A-PB450-A
C
K7M2
U2OS
G2
S
100
100
10.8
8.69
7.29
G1
19.6
20.3
20.8
80
80
45.5
48.6
42.0
60
60
Cell number (%)
41.7
40.6
45.5
40
40
20
20
41.9
40.6
49.3
36.9
37.3
31.6
0
0
Control
Control
shCtBP2
shCtBP2
OE-CtBP2
OE-CtBP2

## Slide 5
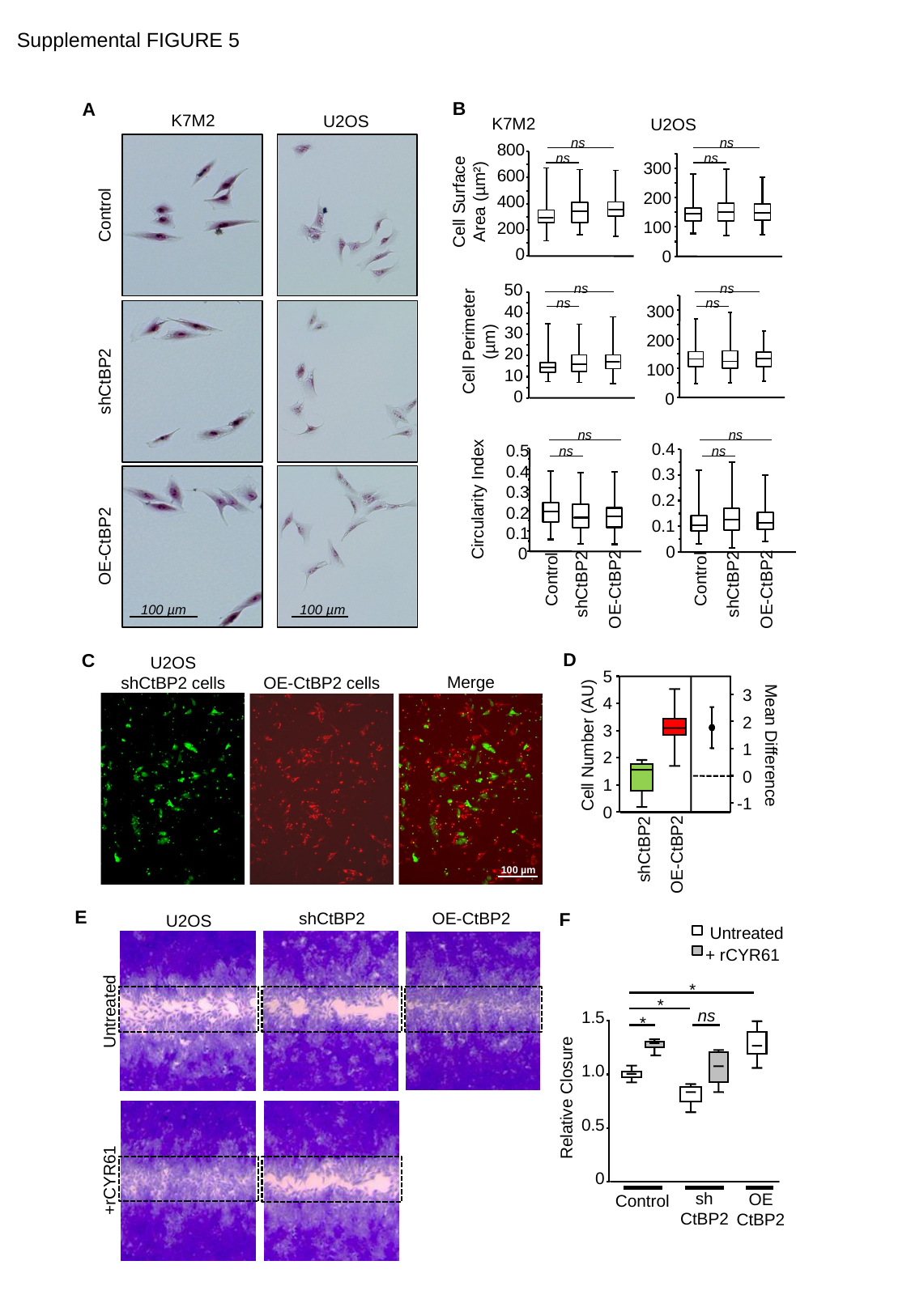

Supplemental FIGURE 5
B
A
K7M2
U2OS
Control
F
shCtBP2
OE-CtBP2
100 µm
100 µm
K7M2
U2OS
ns
ns
800
ns
ns
300
600
Cell Surface
Area (µm²)
200
400
100
200
0
0
ns
ns
50
ns
ns
40
300
30
Cell Perimeter
(µm)
200
20
100
10
0
0
ns
ns
ns
ns
0.4
0.5
0.4
0.3
0.3
0.2
Circularity Index
0.2
0.1
0.1
0
0
Control
Control
shCtBP2
shCtBP2
OE-CtBP2
OE-CtBP2
D
C
U2OS
shCtBP2 cells
Merge
OE-CtBP2 cells
100 µm
5
3
4
2
3
Cell Number (AU)
Mean Difference
1
2
0
1
-1
0
shCtBP2
OE-CtBP2
E
F
shCtBP2
OE-CtBP2
U2OS
Untreated
+rCYR61
Untreated
+ rCYR61
*
*
ns
1.5
*
1.0
Relative Closure
0.5
0
sh
CtBP2
OE
CtBP2
Control

## Slide 6
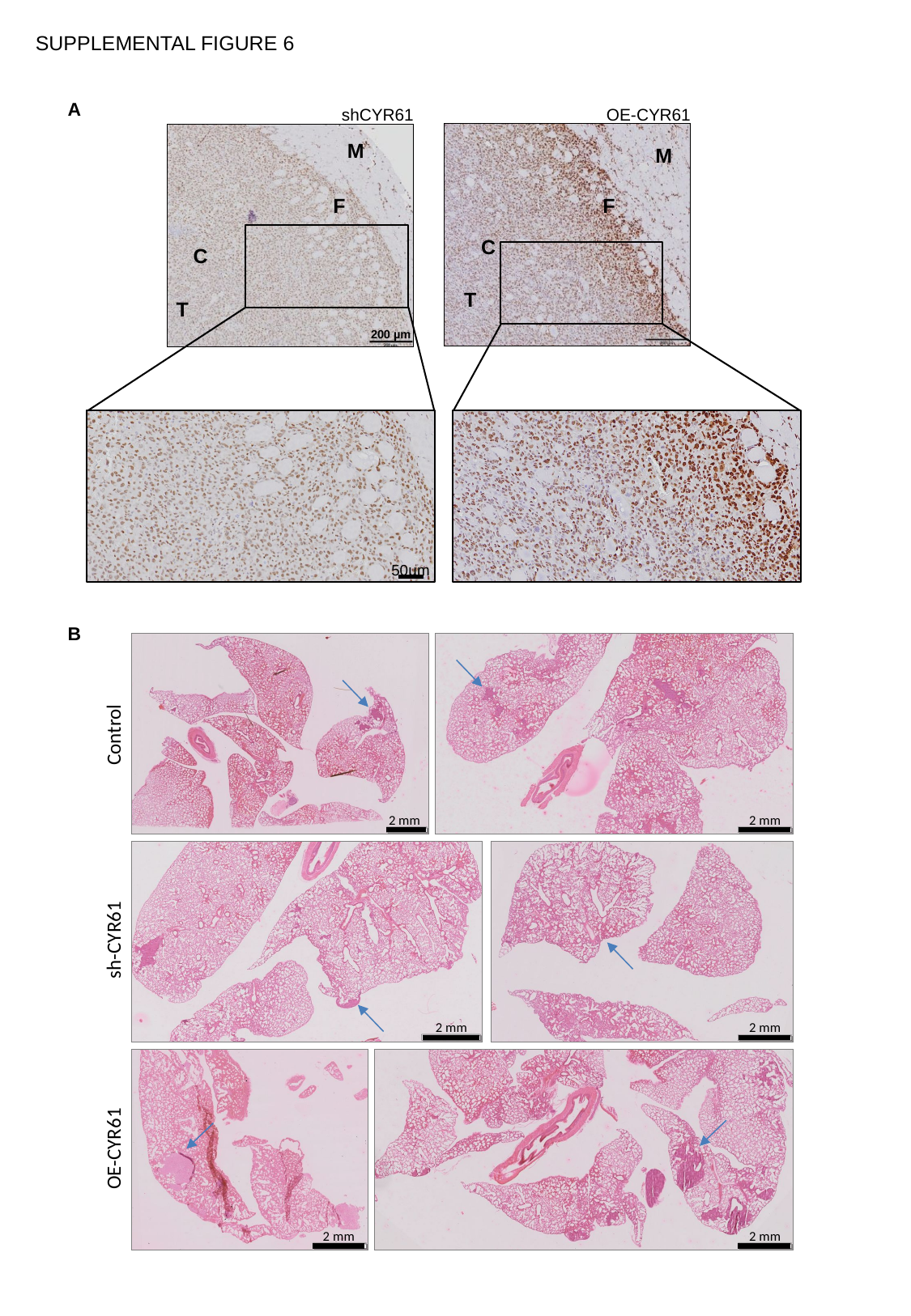

SUPPLEMENTAL FIGURE 6
A
shCYR61
OE-CYR61
M
M
F
F
C
C
T
T
200 µm
50µm
B
Control
2 mm
2 mm
sh-CYR61
2 mm
2 mm
OE-CYR61
2 mm
2 mm
